# Re-evaluation of the comparative effectiveness of bootstrap-based optimism correction methods in the development of multivariable clinical prediction models

**DOI:** 10.1186/s12874-020-01201-w

**Published:** 2021-01-07

**Authors:** Katsuhiro Iba, Tomohiro Shinozaki, Kazushi Maruo, Hisashi Noma

**Affiliations:** 1 Department of Statistical Science, School of Multidisciplinary Sciences, The Graduate University for Advanced Studies, Tokyo, Japan; 2 Office of Biostatistics, Department of Biometrics, Headquarters of Clinical Development, Otsuka Pharmaceutical Co., Ltd., Tokyo, Japan; 3 Department of Information and Computer Technology, Faculty of Engineering, Tokyo University of Science, Tokyo, Japan; 4 Department of Biostatistics, Faculty of Medicine, University of Tsukuba, Ibaraki, Japan; 5 Department of Data Science, The Institute of Statistical Mathematics, 10-3 Midori-cho, Tachikawa, Tokyo, 190-8562 Japan

**Keywords:** Multivariable clinical prediction model, Optimism, *C*-statistic, Regularized estimation, Bootstrap

## Abstract

**Background:**

Multivariable prediction models are important statistical tools for providing synthetic diagnosis and prognostic algorithms based on patients’ multiple characteristics. Their apparent measures for predictive accuracy usually have overestimation biases (known as ‘optimism’) relative to the actual performances for external populations. Existing statistical evidence and guidelines suggest that three bootstrap-based bias correction methods are preferable in practice, namely Harrell’s bias correction and the .632 and .632+ estimators. Although Harrell’s method has been widely adopted in clinical studies, simulation-based evidence indicates that the .632+ estimator may perform better than the other two methods. However, these methods’ actual comparative effectiveness is still unclear due to limited numerical evidence.

**Methods:**

We conducted extensive simulation studies to compare the effectiveness of these three bootstrapping methods, particularly using various model building strategies: conventional logistic regression, stepwise variable selections, Firth’s penalized likelihood method, ridge, lasso, and elastic-net regression. We generated the simulation data based on the Global Utilization of Streptokinase and Tissue plasminogen activator for Occluded coronary arteries (GUSTO-I) trial Western dataset and considered how event per variable, event fraction, number of candidate predictors, and the regression coefficients of the predictors impacted the performances. The internal validity of *C*-statistics was evaluated.

**Results:**

Under relatively large sample settings (roughly, events per variable ≥ 10), the three bootstrap-based methods were comparable and performed well. However, all three methods had biases under small sample settings, and the directions and sizes of biases were inconsistent. In general, Harrell’s and .632 methods had overestimation biases when event fraction become lager. Besides, .632+ method had a slight underestimation bias when event fraction was very small. Although the bias of the .632+ estimator was relatively small, its root mean squared error (RMSE) was comparable or sometimes larger than those of the other two methods, especially for the regularized estimation methods.

**Conclusions:**

In general, the three bootstrap estimators were comparable, but the .632+ estimator performed relatively well under small sample settings, except when the regularized estimation methods are adopted.

**Supplementary Information:**

The online version contains supplementary material available at 10.1186/s12874-020-01201-w.

## Background

Multivariable prediction models have been important statistical tools to provide synthetic diagnosis and prognostic algorithms based on the characteristics of multiple patients [1]. A multivariable prediction model is usually constructed using an adequate regression model (e.g., a logistic regression model for a binary outcome) based on a series of representative patients, but it is well known that their “apparent” predictive performances such as discrimination and calibration calculated in the derivation dataset are better than the actual performance for an external population [2]. This bias is known as “optimism” in prediction models. Practical guidelines (e.g., the Transparent Reporting of a multivariable prediction model for Individual Prognosis Or Diagnosis (TRIPOD) statements [2, 3]) recommend optimism adjustment using principled internal validation methods, e.g., split-sample, cross-validation (CV), and bootstrap-based corrections. Among these validation methods, split-sample analysis is known to provide a relatively imprecise estimate, and CV is not suitable for some performance measures [4]. Thus, bootstrap-based methods are a good alternative because they provide stable estimates for performance measures with low biases [2, 4].

In terms of bootstrapping approaches, there are three effective methods to correct for optimism, specifically Harrell’s bias correction and the .632 and .632+ estimators [1, 5, 6]. In clinical studies, Harrell’s bias correction has been conventionally applied for internal validation, while the .632 and .632+ estimators are rarely seen in practice. This is because the Harrell’s method can be implemented by a relatively simple algorithm (it can be implemented by rms package in R) and the two methods were reported to have similar performances with the Harrell’s method in previous simulation studies [4]. These three estimators are derived from different concepts, and may exhibit different performances under realistic situations. Several simulation studies have been conducted to assess their comparative effectiveness. Steyerberg et al. [4] compared these estimators for ordinary logistic regression models with the maximum likelihood (ML) estimate, and reported that their performances were not too different under relatively large sample settings. Further, Mondol and Rahman [7] conducted similar simulation studies to assess their performances under rare event settings in which event fraction is about 0.1 or lower. They also considered Firth’s penalized likelihood method [8, 9] for estimating regression coefficients, and concluded that the .632+ estimator performed especially well. Several other modern effective estimation methods have been widely adopted in clinical studies. Representative approaches include regularized estimation methods such as ridge [10], lasso (least absolute shrinkage and selection operator) [11], and elastic-net [12]. Also, conventional stepwise variable selections are still widely adopted in current practice [13]. Note that the previous studies of these various estimation methods did not assess the comparative effectiveness of the bootstrapping estimators. Also, their simulation settings were not very comprehensive because their simulations were conducted to assess various methods and outcome measures, and comparisons of the bootstrap estimators comprised only part of their analyses.

In this work, we conducted extensive simulation studies to provide statistical evidence concerning the comparative effectiveness of the bootstrapping estimators, as well as to provide recommendations for their practical use. In particular, we evaluated these methods using multivariable prediction models that were developed with various model building strategies: conventional logistic regression (ML), stepwise variable selection, Firth’s penalized likelihood, ridge, lasso, and elastic-net. We considered extensive simulation settings based on a real-world clinical study data, the Global Utilization of Streptokinase and Tissue plasminogen activator for Occluded coronary arteries (GUSTO-I) trial Western dataset [14, 15]. Note that we particularly focused on the evaluation of the *C*-statistic [16] in this work, since it is the most popular discriminant measure in clinical prediction models, because the simulation data should be too rich for the extensive studies; the generalizations to other performance measures would be discussed in the Discussion section.

## Methods

### Model-building strategies for multivariable prediction models

At first, we briefly introduce the estimating methods for the multivariable prediction models. We consider constructing a multivariable prediction model for a binary outcome *y*_*i*_ (*y*_*i*_ = 1: event occurring, or 0: not occurring) (*i* = 1, 2, …, *n*). A logistic regression model *π*_*i*_ = 1/(1 + exp(−***β***^*T*^***x***_*i*_)) is widely used as a regression-based prediction model for the probability of event occurrence *π*_*i*_ = Pr(*y*_*i*_ = 1| ***x***_*i*_) [2, 17]. ***x***_*i*_ = (1, *x*_*i*1_, *x*_*i*2_, …, *x*_*ip*_)^*T*^ (*i* = 1, 2, …, *n*) are *p* predictor variables for an individual *i* and ***β*** = (*β*_0_, *β*_1_, …, *β*_*p*_)^*T*^ are the regression coefficients containing the intercept. Plugging an appropriate estimate $\hat{\boldsymbol{\beta}}$ of ***β*** into the above model, the estimated probability ${\hat{\pi}}_i$ (*i* = 1, 2, …, *n*) is defined as the risk score of individual patients, and this score is used as the criterion score to determine the predicted outcome.

The ordinary ML estimation can be easily implementable by standard statistical software and has been widely adopted in practice [2, 17]. However, the ML-based modelling strategy is known to have several finite sample problems (e.g., overfitting and (quasi-)complete separation) when applied to a small or sparse dataset [9, 18–20]. Both properties disappear with increasing events per variables (EPVs), defined as the ratio of the number of events to the number of predictor variables (*p*) of the prediction model. EPV has been formally adopted as a sample size criterion for model development, and in particular, EPV ≥ 10 is a widely adopted criterion [21]; recent studies showed that the validity of this criterion depended on case-by-case conditions [4, 13, 22]. As noted below, the following shrinkage estimation methods can moderate these problems.

For variable selection, backward stepwise selection has been generally recommended for the development of prediction models [23]. For the stopping rule, the significance level criterion (a conventional threshold is *P* < .050), Akaike Information Criterion (AIC) [24], and Bayesian Information Criterion (BIC) [25] can be adopted. Regarding AIC and BIC, there is no certain evidence which criterion is absolutely better in practices. However, several previous studies have reported AIC was more favorable [23, 26, 27], and thus we adopted only AIC in our simulation studies.

Several shrinkage estimation methods such as Firth’s logistic regression [8], ridge [10], lasso [11], and elastic-net [12] have been proposed. These shrinkage methods estimate the regression coefficients based on penalized log likelihood function. These methods can deal with (quasi-)complete separation problem [9, 17]. Also, since the estimates of the regression coefficients are shrunk towards zero, these methods can alleviate overfitting [17, 28, 29]. Lasso and elastic-net can shrink some regression coefficients to be exactly 0; therefore, lasso and elastic-net can perform shrinkage estimation and variable selection simultaneously [11, 12]. The penalized log likelihood function of ridge, lasso, and elastic-net include a turning parameter to control the degree of shrinkage [10–12]. Elastic-net also has a turning parameter to determine the weight of lasso type and ridge type penalties [12]. Several methods, such as CV, were proposed for selection of the tuning parameters of ridge, lasso, and elastic-net [17, 28, 29].

In numerical analyses in the following sections, all of the above methods were performed using R version 3.5.1 language programming (The R Foundation for Statistical Computing, Vienna, Austria) [30]. The ordinary logistic regression was fitted by the glm function. The stepwise variable selections were performed using the stats and logistf packages [31]. Firth’s logistic regression was also conducted using the logistf package [31]. The ridge, lasso, and elastic-net regressions were performed using the glmnet package [32]; the turning parameters were consistently determined using the 10-fold CV of deviance.

### C-statistic and the optimism-correction methods based on bootstrapping

#### C-statistic

Secondly, we review the internal validation methods used in the numerical studies. We focused specifically on the *C*-statistic in our numerical evaluations, as it is most frequently used in clinical studies as an summary measure of the discrimination of prediction models [2]. The *C*-statistic is defined as the empirical probability that a randomly selected patient who has experienced an event has a larger risk score than a patient who has not experienced the event [16]. The *C*-statistic also corresponds to a nonparametric estimator of the area under the curve (AUC) of the empirical receiver operating characteristic (ROC) curve for the risk score. The *C*-statistic ranges from 0.5 to 1.0, with larger values corresponding to superior discriminant performance.

#### Harrell’s bias correction method

The most widely applied method for bootstrapping optimism correction is Harrell’s bias correction [1], which is obtained by the conventional bootstrap bias correction method [4]. The algorithm is summarized as follows:

- Let *θ*_*app*_ be the apparent predictive performance estimate for the original population.
- Generate *B* bootstrap samples by resampling with replacement from the original population.
- Construct a prediction model for each bootstrap sample, and calculate the predictive performance estimate for it. We denote the *B* bootstrap estimates as *θ*_1, *boot*_, *θ*_2, *boot*_, ⋯, *θ*_*B*, *boot*_.
- Using the *B* prediction models constructed from the bootstrap samples, calculate the predictive performance estimates for the original population: *θ*_1, *orig*_, *θ*_2, *orig*_, ⋯, *θ*_*B*, *orig*_.
- The bootstrap estimate of optimism is obtained as
  $$ \Lambda =\frac{1}{B}\sum \limits_{b=1}^B\left({\theta}_{b, boot}-{\theta}_{b, orig}\right) $$

Subtracting the estimate of optimism from the apparent performance, the bias corrected predictive performance estimate is obtained as *θ*_*app*_ − Λ.

The bias correction estimator is calculable by a relatively simple algorithm, and some numerical evidence has shown that it performs well under relatively large sample settings (e.g., EPV ≥ 10) [4]. Therefore, this algorithm is currently adopted in most clinical prediction model studies that conduct bootstrap-based internal validations. However, a certain proportion of patients in the original population (on average, 63.2%) should be overlapped in the bootstrap sample [6]. The overlap may cause overestimation of the predictive performance [27], and several alternative estimators have therefore been proposed, as follows.

#### Efron’s .632 method

Efron’s .632 method [5] was proposed as a bias-corrected estimator that considers overlapped samples. Among the *B* bootstrap samples, we consider the “external” samples that are not included in the bootstrap samples to be ‘test’ datasets for the developed *B* prediction models. Then, we calculate the predictive performance estimates for the external samples by the developed *B* prediction models *θ*_1, *out*_, *θ*_2, *out*_, ⋯, *θ*_*B*, *out*_, and we denote the average measure as ${\theta}_{out}={\sum}_{b=1}^B{\theta}_{b, out}/B$. Thereafter, the .632 estimator is defined as a weighted average of the predictive performance estimate in the original sample *θ*_*app*_ and the external sample estimate *θ*_*out*_:

$$ {\theta}_{.632}=0.368\times {\theta}_{app}+0.632\times {\theta}_{out} $$

The weight .632 derives from the approximate proportion of subjects included in a bootstrap sample. Since the subjects that are included and not included in a bootstrap sample are independent, the .632 estimator can be interpreted as an extension of CV [4, 7]. However, the .632 estimator is associated with overestimation bias under highly overfit situations, when the apparent estimator *θ*_*app*_ has a large bias [6].

#### Efron’s .632+ method

Efron and Tibshirani [6] proposed the .632+ estimator to address the problem of the .632 estimator by taking into account the amount of overfitting. They define the relative overfitting rate *R* as

$$ R=\frac{\theta_{out}-{\theta}_{app}}{\gamma -{\theta}_{app}} $$

*γ* corresponds to ‘no information performance’, which is the predictive performance measure for the original population when the outcomes are randomly permuted. Although the *γ* can be set to an empirical value, we used *γ* = 0.50 that is the theoretical value for the case of the *C*-statistic [4]. The overfitting rate *R* approaches 0 when there is no overfitting (*θ*_*out*_ = *θ*_*app*_), and approaches 1 when the degree of overfitting is large. Then, the .632+ estimator [6] is defined as

$$ {\theta}_{.632+}=\left(1-w\right)\times {\theta}_{app}+w\times {\theta}_{out} $$

$$ w=\frac{.632}{1-.368\times R} $$

Note that the weight *w* ranges from .632 (*R* = 0) to 1 (*R* = 1). Hence, the .632+ estimator approaches the .632 estimator when there is no overfitting and approaches the external sample estimate *θ*_*out*_ when there is marked overfitting.

In the following numerical studies, the numbers of bootstrap resamples were consistently set to *B* = 2000. Also, for the model-building methods involving variable selections (e.g., stepwise regression) and shrinkage methods which require tuning parameter selections (ridge, lasso, and elastic-net), all estimation processes were included in the bootstrapping analyses in order to appropriately reflect their uncertainty.

### Real-data example: applications to the GUSTO-I trial Western dataset

Since we consider the GUSTO-I trial Western dataset as a model example for the simulation settings, we first illustrate the prediction model analyses for this clinical trial. The GUSTO-I dataset has been adopted by many performance evaluation studies of multivariable prediction models [4, 13, 29], and we specifically used the West region dataset [23]. GUSTO-I was a comparative clinical trial to assess four treatment strategies for acute myocardial infarction [14]. Here we adopted death within 30 days as the outcome variable (binary). The summary of the outcome and the 17 covariates are presented in Table 1. Of the 17 covariates, two variables (height and weight) are continuous, one variable (smoking) is ordinal, and the remaining 14 variables are binary; age was dichotomized at 65 years old. For smoking, which is a three-category variable (current smokers, ex-smokers, and never smokers), we generated two dummy variables (ex-smokers vs. never smokers, and current smokers vs. never smokers) and used them in the analyses.

**Table 1:** Characteristics of the GUSTO-I Western dataset

N	2188
Outcome	
30-day mortality	6.2%
Covariates	
Age > 65 years	38.4%
Female gender	24.9%
Diabetes	14.3%
Hypotension (systolic blood pressure < 100 mmHg)	9.6%
Tachycardia (pulse > 80 bpm)	33.4%
High risk (anterior infarct location/previous MI)	48.7%
Shock (Killip class III/IV)	1.5%
Time to relief of chest pain > 1 h	60.9%
Previous MI	17.1%
Height (cm)	172.1 ± 10.1
Weight (kg)	82.9 ± 17.7
Hypertension history	40.4%
Ex-smoker	30.8%
Current smoker	27.9%
Hypercholesterolemia	40.5%
Previous angina pectoris	34.1%
Family history of MI	47.6%
ST elevation in > 4 leads	35.6%

We considered two modelling strategies: (1) 8-predictor models (age > 65 years, female gender, diabetes, hypotension, tachycardia, high risk, shock, and no relief of chest pain), which was adopted in several previous studies [4, 33]; and (2) 17-predictor models which included all variables presented in Table 1. The EPVs for these models were 16.9 and 7.5, respectively. For the two modelling strategies, we constructed multivariable logistic prediction models using the estimating methods in the Methods section (ML, Firth, ridge, lasso, elastic-net) and backward stepwise methods with AIC and statistical significance (*P* < 0.05). We also calculated the *C*-statistics and the bootstrap-based optimism-corrected estimates for these prediction models. The results are presented in Table 2.

**Table 2:** Regression coefficients estimates and predictive performance measures for the GUSTO-I trial Westen dataset

	8-Predictor Model							17-Predictor Model						
	ML	Firth	Ridge	Lasso	Elastic-net	Backward (AIC)	Backward (*P* < 0.05)	ML	Firth	Ridge	Lasso	Elastic-net	Backward (AIC)	Backward (*P* < 0.05)
Coefficients:														
Intercept	−5.092	−5.034	−4.787	−4.933	− 4.886	− 4.927	− 4.927	−5.090	−4.983	−3.434	−3.494	−3.486	−3.494	−2.853
Age > 65 years	1.637	1.616	1.424	1.578	1.535	1.631	1.631	1.429	1.399	1.161	1.336	1.324	1.495	1.532
Female gender	0.622	0.620	0.592	0.586	0.586	0.624	0.624	0.490	0.487	0.380	0.288	0.289	0.368	.
Diabetes	0.069	0.083	0.078	0.024	0.035	.	.	0.153	0.164	0.131	.	.	.	.
Hypotension	1.218	1.215	1.102	1.164	1.145	1.252	1.252	1.192	1.178	1.037	1.036	1.030	1.230	1.227
Tachycardia	0.650	0.645	0.574	0.608	0.597	0.661	0.661	0.653	0.643	0.533	0.530	0.526	0.669	0.717
High risk	0.847	0.835	0.748	0.796	0.781	0.855	0.855	0.403	0.397	0.390	0.372	0.372	0.414	2.748
Shock	2.395	2.362	2.339	2.362	2.354	2.424	2.424	2.685	2.608	2.508	2.460	2.455	2.662	0.779
No relief of chest pain	0.263	0.255	0.237	0.219	0.221	.	.	0.233	0.223	0.200	0.107	0.107	.	.
Previous MI								0.505	0.495	0.437	0.378	0.376	0.586	.
Height								0.008	0.008	−0.001	.	.	.	.
Weight								−0.019	−0.018	−0.015	−0.014	−0.014	−0.018	−0.023
Hypertension history								−0.165	−0.159	−0.123	.	.	.	.
Ex-smoker								0.174	0.169	0.147	.	.	.	.
Current smoker								0.247	0.241	0.231	0.057	0.059	.	.
Hypercholesterolemia								−0.064	−0.060	−0.064	.	.	.	.
Previous angina pectoris								0.246	0.243	0.235	0.151	0.151	.	.
Family history of MI								−0.015	−0.014	−0.036	.	.	.	.
ST elevation in > 4 leads								0.583	0.571	0.479	0.434	0.430	0.601	0.752
*C*-statistics:														
Apparent	0.819	0.819	0.819	0.819	0.819	0.820	0.820	0.832	0.832	0.831	0.831	0.831	0.829	0.824
Harrell	0.810	0.810	0.812	0.810	0.810	0.811	0.810	0.811	0.811	0.812	0.812	0.812	0.810	0.806
.632	0.811	0.811	0.812	0.811	0.810	0.811	0.809	0.811	0.811	0.813	0.813	0.813	0.809	0.808
.632+	0.810	0.811	0.812	0.811	0.810	0.811	0.809	0.810	0.810	0.812	0.812	0.812	0.809	0.808

For the 8-predictor models, the lasso and elastic-net models selected all eight variables. Also, the two backward stepwise methods selected the same six variables for the 8-predictor models; only ‘diabetes’ and ‘no relief of chest pain’ were excluded. Further, for the 17-predictor models, the lasso and elastic-net models selected the same 12 variables, while ‘diabetes’, ‘height’, ‘hypertension history’, ‘ex-smoker’, ‘hypercholesterolemia’, and ‘family history of MI’ were excluded. For the backward stepwise selections, the AIC method selected nine variables and the significance-based method selected seven variables; see Table 2 for the selected variables.

In general, the apparent *C*-statistics of the 17-predictor models were larger than those of the 8-predictor models. Although the apparent *C*-statistics of all the prediction models were comparable for the 8-predictor models (about 0.82), the apparent *C*-statistics of the 17-predictor models were ranged from 0.82 to 0.83. The optimism-corrected *C*-statistics (about 0.81) were smaller than the apparent *C*-statistics for all models, and certain biases were discernible. However, among the three bootstrapping methods, there were no differences for any of the prediction models. Note that the optimism-corrected *C*-statistics between the 8- and 17-predictor models were comparable. These results indicate that the 17-predictor model had greater optimism, possibly due to the inclusion of noise variables.

### Simulations

#### Data generation and simulation settings

As described in the previous section, we conducted extensive simulation studies to assess the bootstrap-based internal validation methods based on a real-world dataset, the GUSTO-I Western dataset. We considered a wide range of conditions with various factors that can affect predictive performance: the EPV (3, 5, 10, 20, and 40), the expected event fraction (0.5, 0.25, 0.125, and 0.0625), the number of candidate predictors (eight variables, as specified in the previous studies, and all 17 variables), and the regression coefficients of the predictor variables (two scenarios, as explained below). All combinations of these settings were covered, and a total of 80 scenarios were investigated. The settings of the EPV and the event fraction were based on those used in previous studies [4, 17]. For the regression coefficients of the predictor variables (except for the intercept *β*_0_), we considered two scenarios: one fixed to the ML estimate for the GUSTO-I Western dataset (scenario 1) and the other fixed to the elastic-net estimate for the GUSTO-I Western dataset (scenario 2). In scenario 1, all the predictors have some effect on the risk of events, while in scenario 2 some of the predictor effects are null and the others are relatively small compared with scenario 1. The intercept *β*_0_ was set to properly adjust the event fractions. The sample size of the derivation cohort *n* was determined by (the number of candidate predictor variables × EPV) / (expected event fraction).

The predictor variables were generated as random numbers based on the parameters estimated from the GUSTO-I Western dataset. Three continuous variables (height, weight, and age) were generated from a multivariate normal distribution with the same mean vector and covariance matrix as in the GUSTO-I Western data; the age variable was dichotomized at age 65 years, similar to the analyses in the real-data example. For smoking, an ordinal variable, random numbers were generated from multinomial distribution using the same proportions as in the GUSTO-I Western data; this variable was converted to two dummy variables before being incorporated into the prediction models. In addition, the remaining binary variables were generated from a multivariate binomial distribution [34] using the same marginal probabilities and correlation coefficients estimated from the GUSTO-I Western dataset. We used the mipfp package [35] for generating the correlated binomial variables. The event occurrence probability *π*_*i*_ (*i* = 1, 2, …, *n*) was determined based on the generated predictor variables ***x***_*i*_ and the logistic regression model *π*_*i*_ = 1/(1 + exp(−***β***^*T*^***x***_*i*_)). The outcome variable *y*_*i*_ was generated from a Bernoulli distribution with a success probability *π*_*i*_.

The actual prediction performances of the developed models were assessed by 500,000 independently generated external test samples; the empirical *C*-statistics for the test datasets, which we refer to as the ‘external’ *C*-statistics, were used as the estimands. The number of simulations was consistently set to 2000 for all the scenarios. Based on the generated derivation cohort dataset, the multivariable prediction models were constructed using the seven modelling strategies (ML, Firth, ridge, lasso, elastic-net, and backward stepwise selections with AIC and *P* < 0.05). The *C*-statistics for the derivation cohort were estimated by the apparent, Harrell, .632, and .632+ bootstrapping methods in the derivation cohort. For the evaluation measures, biases and RMSEs (root mean squared errors) for the estimated *C*-statistics from the external *C*-statistic for the 500,000 test datasets were used.

In order to assess sensitivity to involve skewed continuous variables as predictors, the three continuous variables were also generated from a multivariate skew normal distribution [36] with the parameters estimated from the GUSTO-I Western dataset. The sensitivity analyses for the skewed variables settings were conducted only for the ML estimation.

#### Simulation results

In the main Tables and Figures, we present the results of scenario 2 with event fractions of 0.5 and 0.0625; as mentioned below, the overall trends were not very different, and these scenarios are representative of the all simulation results. Other simulation results are provided in e-Appendix A in Additional file 1 because the contents were too large to present in this manuscript. In the small-sample settings (low EPVs and large event fractions), the simulation results for modelling strategies involving variable selections (lasso, elastic-net, and stepwise selections) occasionally dropped all of the predictors; in such cases, only an intercept remained, yielding a nonsensical prediction model. We excluded these cases from the performance evaluations because such models are not usually adopted in practice. As reference information, the frequencies with which the intercept models occurred are presented in e-Table 1 in e-Appendix B in Additional file 1. The results of the sensitivity analyses for skewed variables settings are presented in e-Appendix C in Additional file 1.

**Fig. 1:**
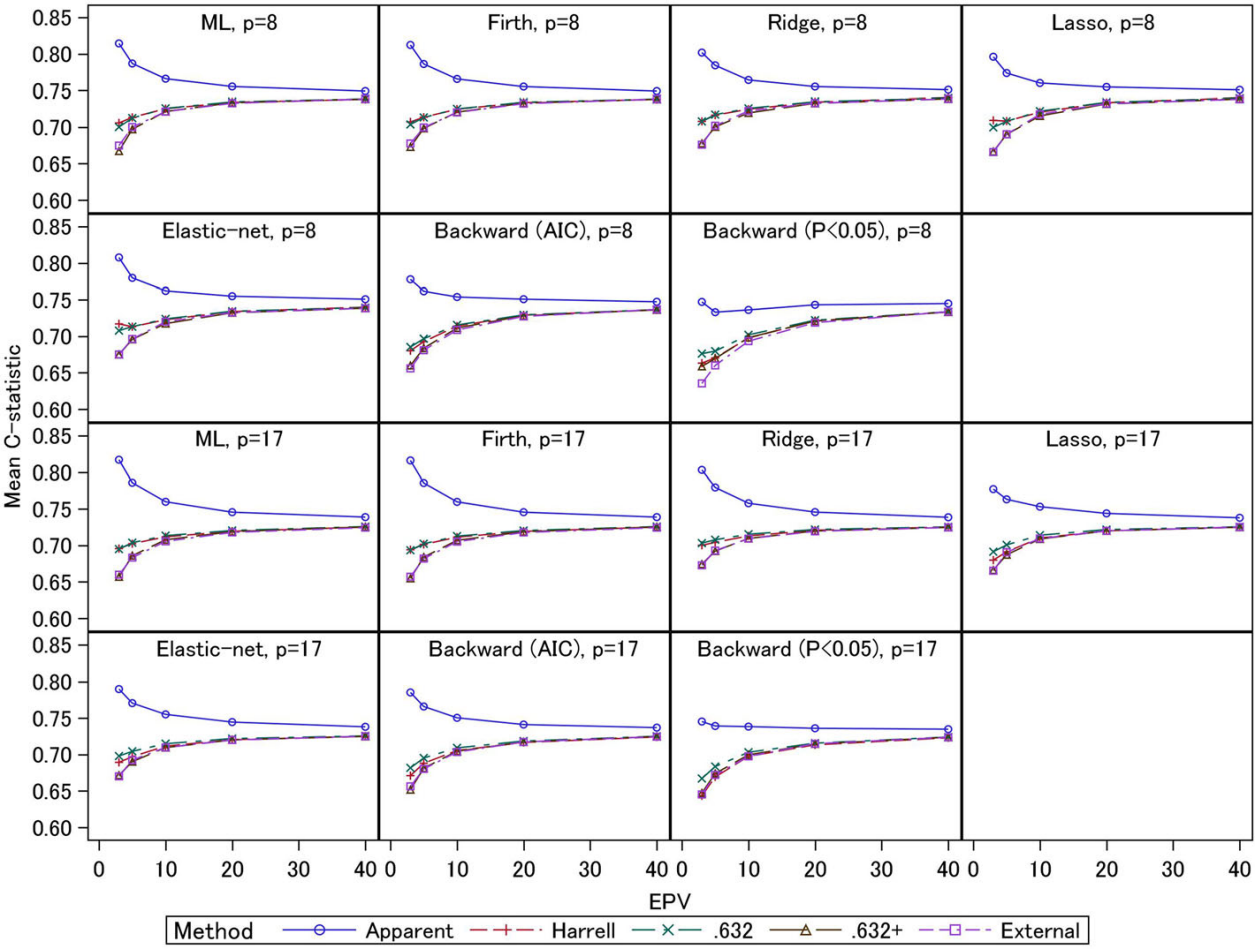
Simulation results: apparent, external, and optimism-corrected *C*-statistics (scenario 2 and event fraction = 0.5)

**Fig. 2:**
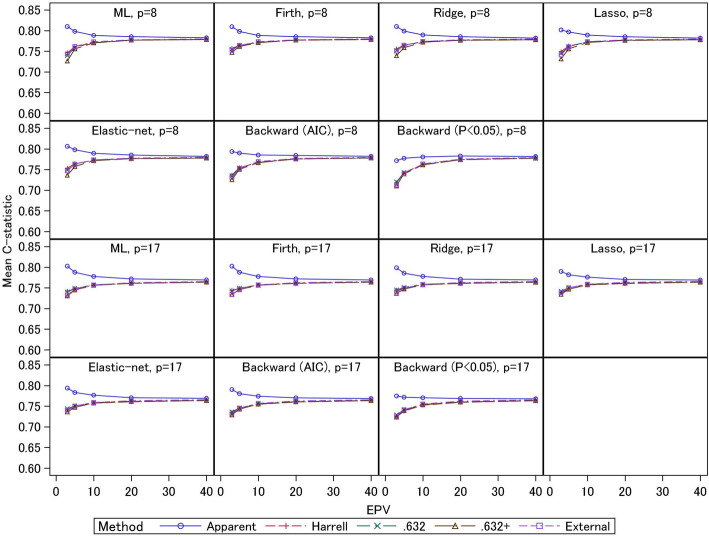
Simulation results: apparent, external, and optimism-corrected *C*-statistics (scenario 2 and event fraction = 0.0625)

The average values of the apparent, external, and optimism-corrected *C*-statistics for 2000 simulations are shown in Fig. 1 (for event fraction = 0.5) and Fig. 2 (for event fraction = 0.0625). Under event fraction = 0.5, the external *C*-statistics for the test datasets were 0.65–0.70 for EPV = 3 and 5, and around 0.72 for larger EPV. These results were similar between the 8- and 17-predictor models. For the 17-predictor models under EPV = 3, the ridge, lasso, and elastic-net showed large external *C*-statistics (0.67) compared with the other models. The external *C*-statistics for the ML estimation, Firth regression, and stepwise selection (AIC) were comparable (0.66). The stepwise selection (*p*< 0.05) had the smallest external *C*-statistic (0.65). For the 8-predictor models under EPV = 3, the external *C*-statistics for the ridge, elastic-net, and Firth regression were comparable (0.68). However, the external *C*-statistic for the lasso was similar to that for the ML estimation (0.67). Both stepwise selections had small external *C*-statistics (0.64–0.66) compared with the other models. In general, the shrinkage methods showed better actual predictive performances than the other methods. In particular, for the 17-predictor models with the noise variables, the ridge, lasso, and elastic-net had better predictive performances compared with the Firth method. However, the lasso did not perform well for the 8-predictor models. This might be caused by total sample size; the scenarios for the 8-predictor models had smaller total sample size. Although the Firth regression showed better predictive performance compared with the ML estimation for the 8-predictor models, the predictive performances of the Firth and ML methods were comparable for the 17-predictor models. Further, the predictive performances of stepwise selections were generally worse than the other methods, as shown by previous studies [17]. Similar trends were observed under event fraction = 0.0625, but the external *C*-statistics (around 0.75, under EPV = 3–5) were larger than those under event fraction = 0.5. These results were caused by the total sample sizes: the event fraction of the latter scenario was smaller and the total sample sizes were larger if EPV were the same. Comparing the modelling strategies, the similar trends as those under event fraction = 0.5 were observed.

**Fig. 3:**
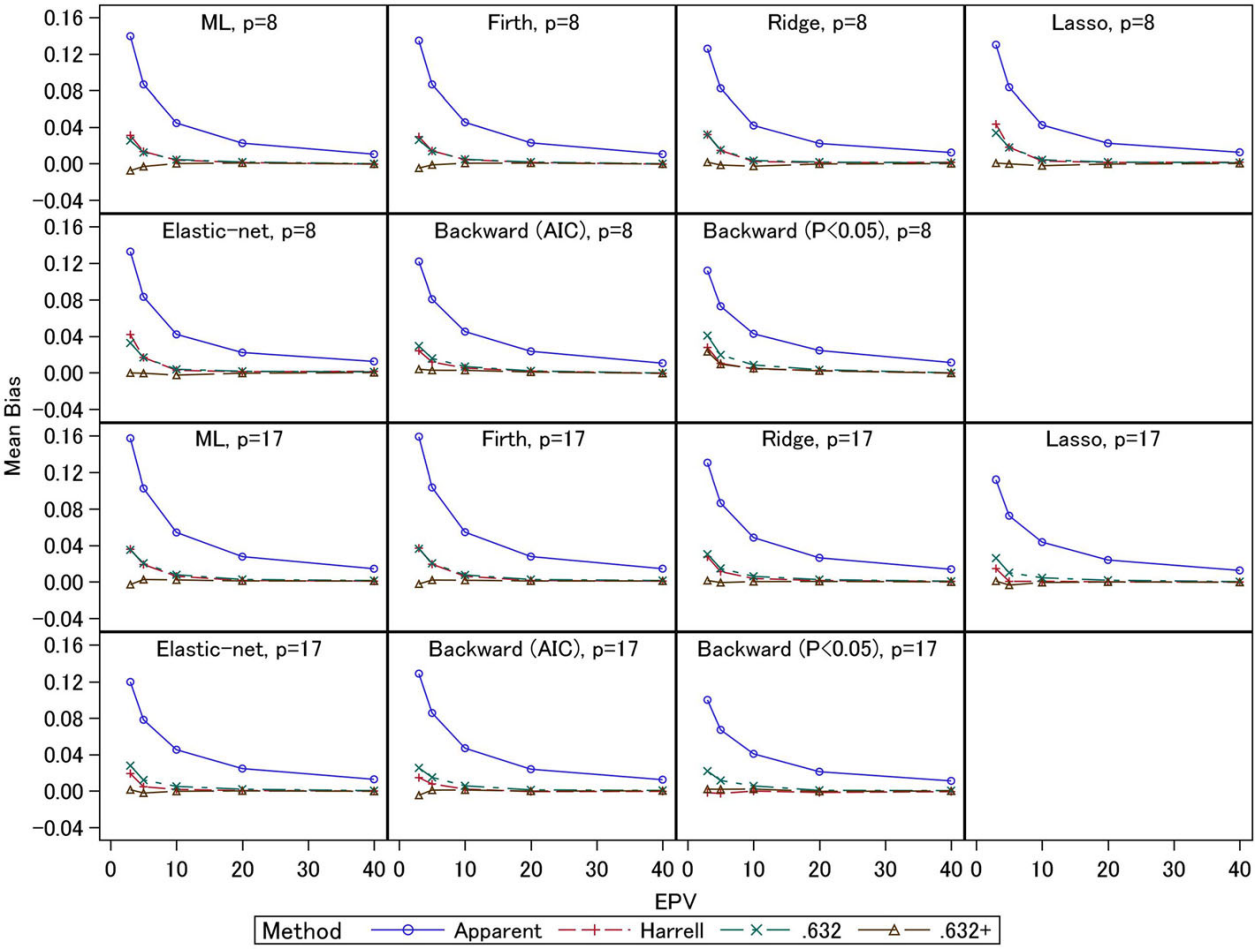
Simulation results: bias in the apparent and optimism-corrected *C*-statistics (scenario 2 and event fraction = 0.5)

**Fig. 4:**
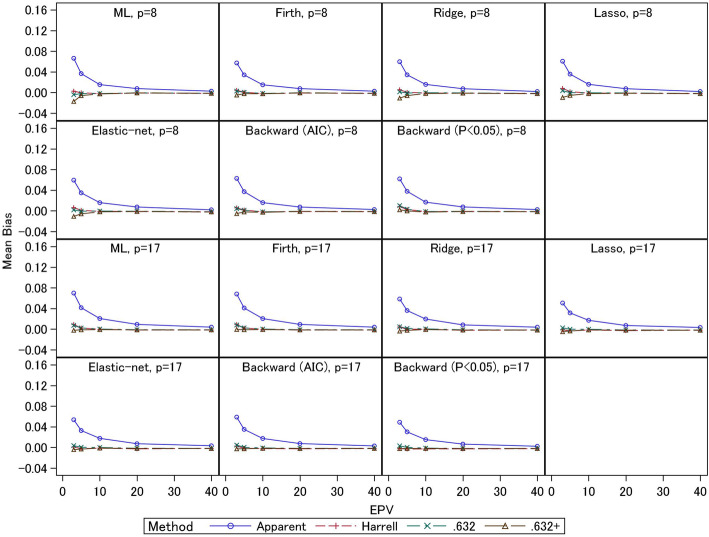
Simulation results: bias in the apparent and optimism-corrected *C*-statistics (scenario 2 and event fraction = 0.0625)

In Figs. 3 and 4, we present the empirical biases of the estimators of *C*-statistics derived from the external *C*-statistics. Under EPV = 3 and 5, the apparent *C*-statistics had large overestimation biases (0.07–0.16 for event fraction = 0.5 and 0.03–0.07 for event fraction = 0.0625) compared with the three bootstrapping methods. In particular, under smaller EPV settings, the overestimation biases were larger. For the same EPV settings, the overestimation biases were smaller when the event fraction was smaller (i.e., the total sample sizes were larger). For 17-predictor models under event fraction = 0.5 and EPV = 3, the overestimation biases of the apparent *C*-statistics of the ML and Firth methods (0.16) were larger than those of the other methods. The ridge and AIC method had large overestimation biases (0.13) compared with the elastic-net and lasso (0.11–0.12). The *P* < 0.05 criterion had the smallest overestimation bias (0.10). For 8-predictor models, the ML estimation showed large overestimation bias (0.14) compared with the other methods. The overestimation biases of the shrinkage methods were comparable (0.13). The stepwise selections had small overestimation biases (0.11–0.12) compared with other methods, but the external *C*-statistics were also smaller, as noted above. Generally, similar trends were observed for event fraction = 0.0625. Comparing the three bootstrapping methods under EPV ≥ 20, the biases of all the methods were comparable for all settings. The results for the conventional ML estimation were consistent with those of Steyerberg et al. (2001) [4], and we confirmed that similar results were obtained for the shrinkage estimation methods. Further, under small EPV settings, unbiased estimates were similarly obtained by the three bootstrapping methods for the 8-predictor models with event fraction = 0.0625, since the total sample size was relatively large, and similar trends were observed for all estimation methods under EPV ≥ 5. Under EPV = 3, the .632+ estimator had underestimation biases, while for the ML estimation, the underestimation bias was 0.02. For ridge, lasso, and elastic-net, the underestimation biases were 0.01. For the Firth regression and stepwise methods, the underestimation biases were less than 0.01. The Harrell and .632 estimators were comparable, and they had overestimation biases (0.01 or lower). For the 17-predictor models, the underestimation biases of the .632+ estimator were less than 0.01, but in general this estimator displayed underestimation biases. The Harrell and .632 estimators had overestimation biases (less than 0.01). Under event fraction = 0.5, the overestimation biases of the Harrell and .632 estimators were remarkably large under EPV = 3 and 5; under the 8-predictor models, the overestimation biases were 0.03–0.04. Although the .632+ estimator had underestimation biases for the ML and Firth methods (0.01 under EPV = 3), mostly unbiased estimates were obtained for the ridge, lasso, and elastic-net estimators. For the stepwise selections, the AIC method provided mostly unbiased estimator, but the *P* < 0.05 criterion resulted in overestimation bias (0.02). For the 17-predictor models, similar trends were observed. The Harrell and .632 estimators had overestimation biases (0.02–0.04 under EPV = 3), and the two estimators were comparable for the ML, Firth, and ridge estimators. However, for the lasso, elastic-net, and stepwise (AIC) methods, whereas the overestimation biases of the Harrell estimator were 0.02, those of the .632 estimator were 0.03. For the stepwise selection method (*P* < 0.05), the .632 estimator showed overestimation bias (0.02), but the Harrell estimator was mostly unbiased. Further, the .632+ estimator had underestimation biases (less than 0.01) for the ML, Firth, and stepwise (AIC) methods; this estimator was mostly unbiased for the ridge, lasso, elastic-net, and stepwise (*P* < 0.05) methods.

**Fig. 5:**
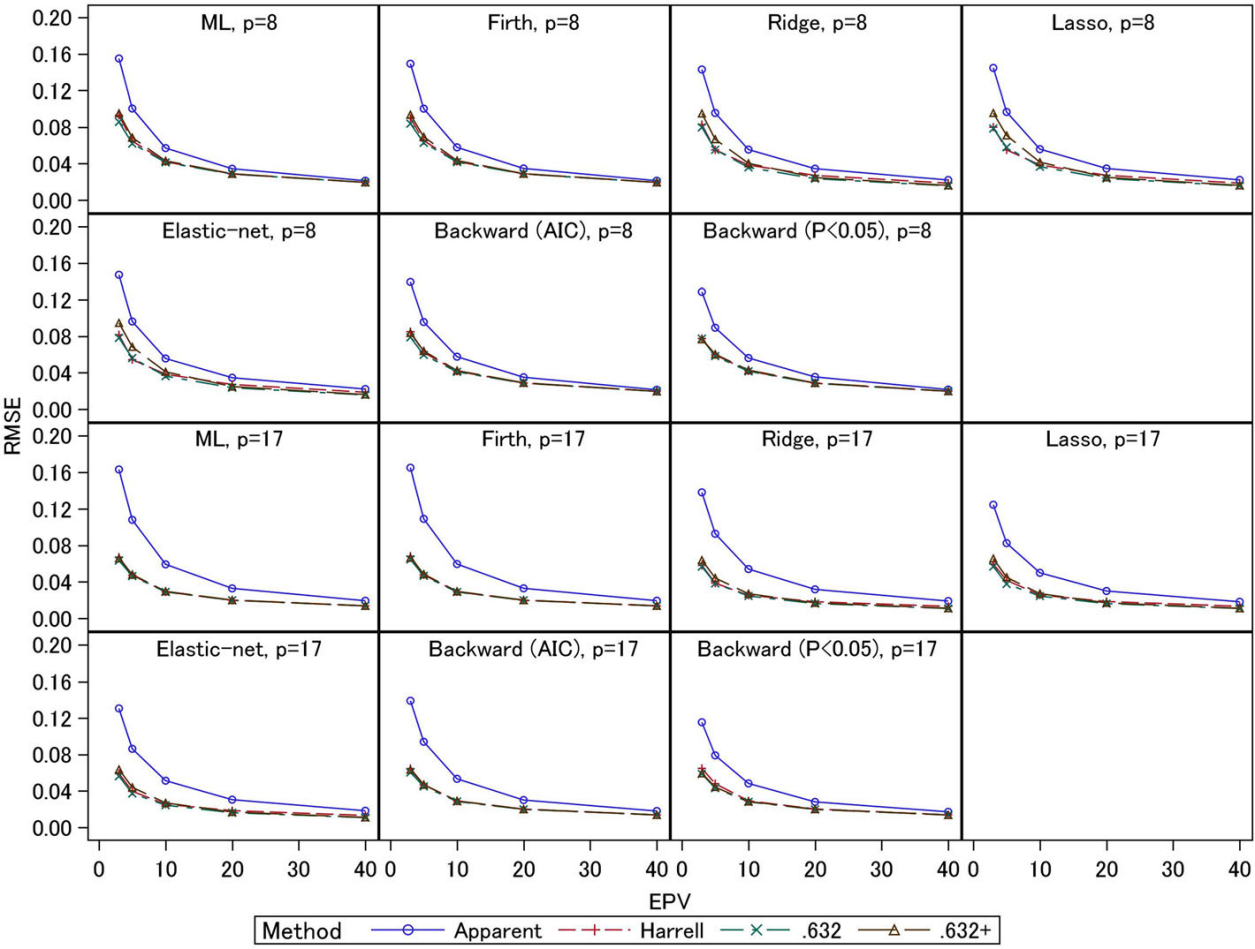
Simulation results: RMSE in the apparent and optimism-corrected *C*-statistics (scenario 2 and event fraction = 0.5)

**Fig. 6:**
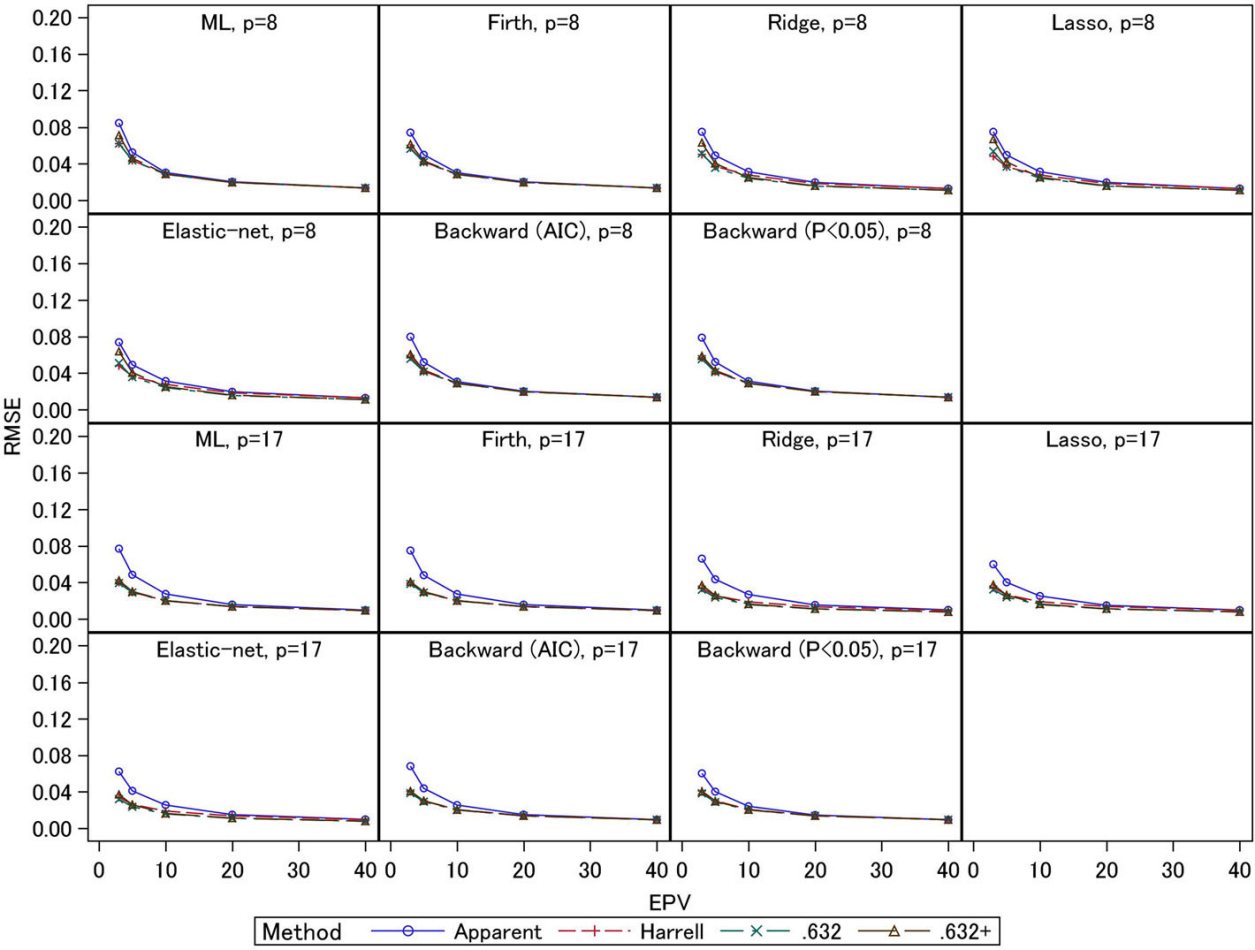
Simulation results: RMSE in the apparent and optimism-corrected *C*-statistics (scenario 2 and event fraction = 0.0625)

The empirical RMSEs are presented in Figs. 5 and 6. Under EPV = 3 and 5, the apparent *C*-statistics had large RMSEs (0.08–0.16 for event fraction = 0.5 and 0.04–0.08 for event fraction = 0.0625) compared with the three bootstrapping methods; these large RMSEs of the apparent *C*-statistics were caused by their large overestimation biases under small EPV, as noted above. The RMSEs of the three bootstrapping methods were generally comparable. An exception is that under event fraction = 0.5 and EPV = 3 and 5, the RMSEs of the .632+ estimators of the 8-predictor models with ridge, lasso, and elastic-net (0.07–0.10) were larger than those of the other two estimators (0.05–0.08). As mentioned above, under these conditions the absolute biases of the .632+ estimators were smaller, reflecting these estimators’ standard errors. For the 17-predictor models, the RMSEs were comparable. Under event fraction = 0.0625, the .632+ estimators for ridge, lasso, and elastic-net had large RMSEs (0.06–0.07) compared with the other two estimators (0.05) for the 8-predictor models under EPV = 3. These results reflect the fact that under these conditions, the absolute biases of the .632+ estimators were larger than those of the other two estimators. In addition, under the other settings with small EPV, there were many scenarios in which the biases of the .632+ estimator were smaller than those of the other two estimators, and in these cases the RMSEs were comparable with those of the Harrell and .632 estimators. These results indicate the .632+ estimator had large standard errors compared with the other two estimators when EPV were small.

The results described above are mostly consistent with those derived under the other settings presented in the e-Appendix A in the Additional file 1.

## Discussion

Bootstrapping methods for internal validations of discriminant and calibration measures in developing multivariable prediction models have been increasingly used in recent clinical studies. The Harrell, .632, and .632+ estimators are asymptotically equivalent (e.g., they have same biases and RMSEs under large sample setting), but in practice they might have different properties in finite sample situations (e.g., the directions and sizes of biases of these estimators are inconsistent in small sample setting). This fact may influence the main conclusions of relevant studies, and conclusive evidence of these estimators’ comparative effectiveness is needed to ensure appropriate research practices. In this work, we conducted extensive simulation studies to assess these methods under a wide range of situations. In particular, we assessed their properties in the context of the prediction models developed by modern shrinkage methods (ridge, lasso, and elastic-net), which are becoming increasingly more popular. We also evaluated stepwise selections, which are additional standard methods of variable selection, taking into consideration the uncertainties of variable selection processes.

Conventionally, the rule-of-thumb criterion for sample size determination in prediction model studies is EPV ≥ 10 [21]. In our simulations, the internal validation methods generally worked well under these settings. However, several counterexamples were reported in previous studies [4, 13, 22], so this should not be an absolute criterion. There were certain conditions in which the standard logistic regression performed well under EPV < 10; relative bias (percentage difference from true value) of the standard logistic regression was 15% or lower [22]. Also, EPV ≥ 10 criterion might not be sufficient for the stepwise selections [13]. The external *C*-statistics of the stepwise selections were smaller than those of ML estimation under certain situations, as previously discussed [13], and variable selection methods might not be recommended in practice. Moreover, the shrinkage regression methods (ridge, lasso, and elastic-net) provided larger *C*-statistics than ML estimation and Firth’s method under certain settings, and were generally comparable. Further investigations are needed to assess the practical value of these methods in clinical studies.

Among the bootstrapping optimism-correction methods, we showed that the Harrell and .632 methods had upward biases at EPV = 3 and 5. The biases in these methods increased when the event fraction became larger. As mentioned in the Methods section, the overlap between the original and bootstrap samples under small sample settings could cause these biases. Therefore, these methods should be used with caution in cases of small sample settings. When the event fraction was 0.5, the .632 estimator often had a greater upward bias than the Harrell method for the shrinkage estimating methods and stepwise selections. Similarly, the .632+ estimator showed upward biases at EPV = 3 for the stepwise selection (*P* < 0.05) for the 8-predictor model. Since the .632+ estimator is constructed by a weighted average of the apparent performance and the out-of-sample performance measures, it cannot have a negative bias when the resultant prediction model has extremely low predictive performance, i.e., when the apparent *C*-statistics are around 0.5. However, if such a prediction model is obtained in practice, we should not adopt it as the final model. Note that these results did not commonly occur in the simulation studies. For example, the cases that the apparent *C*-statistics were less than 0.6 did not been observed for the settings EPV ≥ 10. For the ridge, lasso, elastic-net, and stepwise methods, the frequencies these cases occurred were ranged 0.1–2.1% (median: 0.2%) for EPV = 5 settings, and 0.1–3.6% (median: 0.4%) for EPV = 3 settings.

Also, since the .632+ estimator was developed to overcome the problems of the .632 estimator under highly overfitted situations, the .632+ estimator is expected to have smaller overestimation bias compared with the other two methods. However, the .632+ estimator showed a slight downward bias when the event fraction was 0.0625; the relative overfitting rate was overestimated in that case, since a small number of events was discriminated well by less overfitted models. This tendency was clearly shown for the ML method, which has the strongest overfitting risk.

Although the bias of the .632+ estimator was relatively small, its RMSE was comparable or sometimes larger than those of the other two methods. Since the .632+ estimator adjusts the weights of apparent and out-of-sample performances using the relative overfitting rate, the .632+ estimator has variations due to the variability of the estimated models under small sample settings. Also, the RMSE of the .632+ estimator was particularly large in the shrinkage estimation methods; the penalty parameters were usually selected by 5- or 10-fold CV and we adopted the latter in our simulations. Since the 10-fold CV is unstable with small samples [37], the overfitting rate often has large variations. We attempted to use the leave-one-out CV instead of the 10-fold CV, and this decreased the RMSE of the .632+ estimator (see e-Table 2 in e-Appendix B in Additional file 1). On the other hand, the RMSE in the Harrell method became larger. These results indicate that the performances of the optimism-corrected estimators depend on the methods of penalty parameter selections. Since the external *C*-statistics of lasso using the 10-fold CV and the leave-one-out CV were comparable, the leave-one-out CV showed no clear advantage in terms of penalty parameter selections.

In this work, we conducted simulation studies based on the GUSTO-I study Western dataset. We considered a wide range of settings by varying several factors to investigate detailed operating characteristics of the bootstrapping methods. A limitation of the study is that settings for the predictor variables were based only on the case of the GUSTO-I study Western dataset which evaluated mortality in patients with acute myocardial infarction, and these settings were adopted throughout all scenarios. Thus, the results from our simulation studies cannot be generalized to other cases straightforwardly. Also, we considered only the 8- and 17-predictor models that were adopted in many previous studies [4, 13, 29]. Although other scenarios could be considered, the computational burden of the simulations studies was quite enormous; it requires total 4,000,000 iterations (2000 replication × 2000 bootstrap resampling) in each scenario. Considerations of other scenarios or datasets would be further issues in future studies. In addition, we assessed only the *C*-statistic in this study. Other measures such as the Brier score and calibration slope can also be considered for the evaluation of optimism corrections. However, in previous simulation studies, these measures showed similar trends [4]. Also, the partial area under the ROC curve is another useful discriminant measure that can assess the predictive performance within a certain range of interest (e.g., small false positive rate or high true positive rate) [38, 39]. Its practical usefulness for multivariable prediction models has not been well investigated, and extended simulation studies would also be a further issue in future studies.

## Conclusions

In conclusion, under certain sample sizes (roughly, EPV ≥ 10), all of the internal validation methods based on bootstrapping performed well. However, under small sample settings, all the methods had biases. For the ridge, lasso, and elastic-net methods, although the bias of the .632+ estimator was relatively small, its RMSE could become larger than those of the Harrell and .632 estimators. Under small sample settings, the penalty parameter selection strategy should be carefully considered; one possibility is to adopt the leave-one-out CV instead of the 5- or 10-fold CV. For the other estimation methods, the three bootstrap estimators were comparable in general, but the .632+ estimator performed relatively well under certain settings. In addition, developments of new methods to overcome these issues are future issues to be investigated.

## Supplementary Information

**Additional file 1.**

## Data Availability

The GUSTO-I Western dataset is available at http://www.clinicalpredictionmodels.org.

## Abbreviations

CV: Cross-validation
ML: Maximum likelihood
EPV: Events per variable
AIC: Akaike Information Criterion
BIC: Bayesian Information Criterion
AUC: Area under the curve
ROC: Receiver operating characteristic
RMSE: Root mean squared errors
