# Supplementary material for: Re-evaluation of the comparative effectiveness of bootstrap-based optimism correction methods in the development of multivariable clinical prediction models
Source: BMC Med Res Methodol. 2021 Jan 7;21:9. doi: 10.1186/s12874-020-01201-w (PMC7789544; doi:10.1186/s12874-020-01201-w)
Supplement: Supplementary file 1 — Additional file 1. [file 12874_2020_1201_MOESM1_ESM.pdf]

SUPPLEMENTARY MATERIALS for

Re-evaluation of the comparative effectiveness of bootstrap-based optimism correction methods in the development of multivariable clinical prediction models

Katsuhiro Iba[1,2], Tomohiro Shinozaki[3], Kazushi Maruo[4] and Hisashi Noma[5]

[1] *Department of Statistical Science, School of Multidisciplinary Sciences, The Graduate University for Advanced Studies, Tokyo, Japan*

[2] *Office of Biostatistics, Department of Biometrics, Headquarters of Clinical Development, Otsuka Pharmaceutical Co., Ltd., Tokyo, Japan*

[3] *Department of Information and Computer Technology, Faculty of Engineering, Tokyo University of Science, Tokyo, Japan*

[4] *Department of Biostatistics, Faculty of Medicine, University of Tsukuba, Ibaraki, Japan.*

[5] *Department of Data Science, The Institute of Statistical Mathematics, Tokyo, Japan*

e-Appendix A: Simulation results that are not included in the paper

We present the simulation results that are not included in the paper. The average values of the apparent, external, and optimism-corrected *C*-statistics are presented in e-Figure 1 to e-Figure 4 (scenario 1 with event fractions of 0.5, 0.25, 0.125, and 0.0625) and e-Figure 5 to e-Figure 6 (scenario 2 with event fractions of 0.25 and 0.125). The empirical biases of the estimators of *C*-statistics derived from the external *C*-statistics are presented in e-Figure 7 to e-Figure 12. The empirical RMSEs are presented in e-Figure 13 to e-Figure 18.

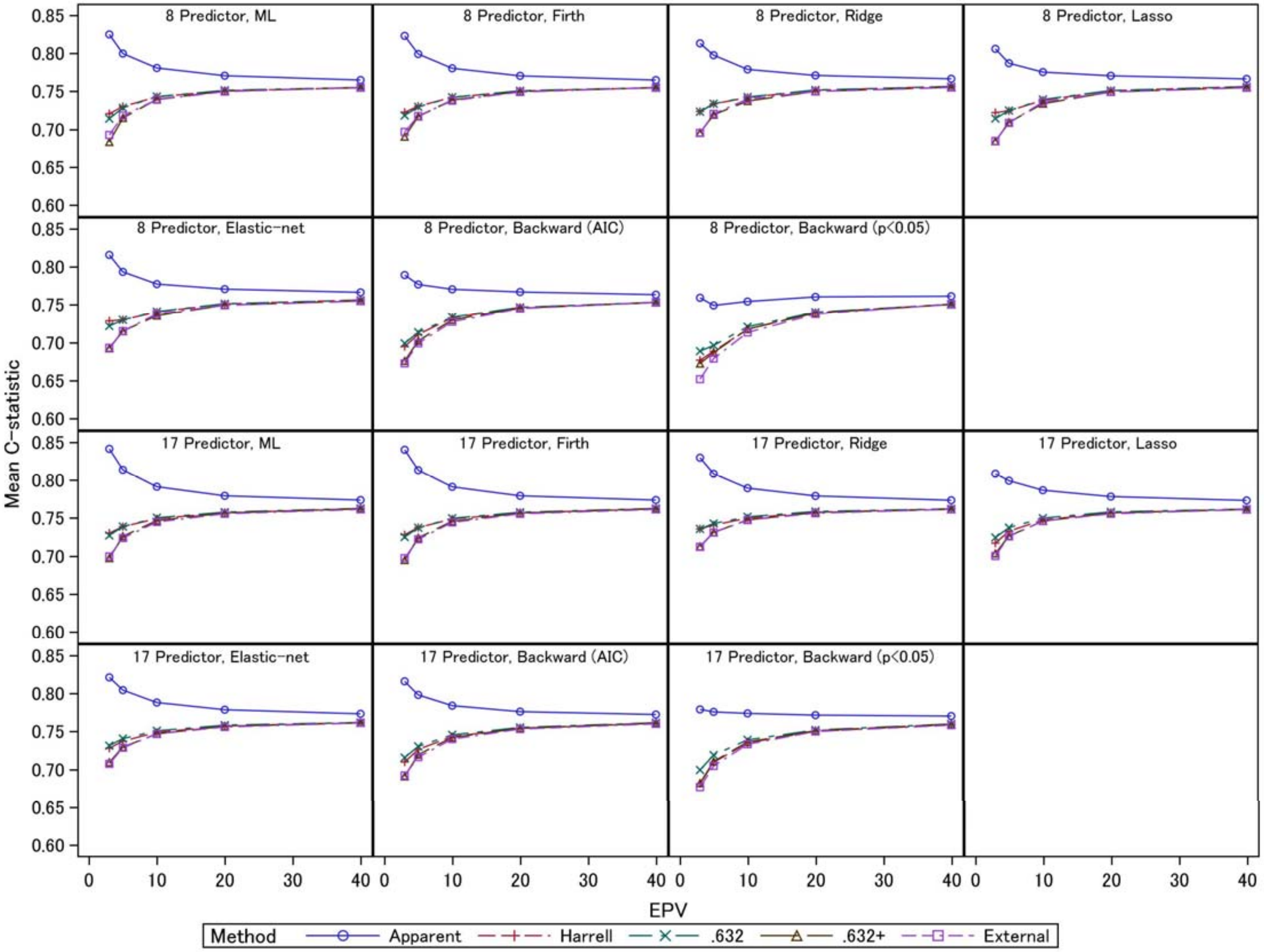

e-Figure 1. Simulation results: apparent, external, and optimism corrected *C*-statistics (scenario 1 and event fraction = 0.5)

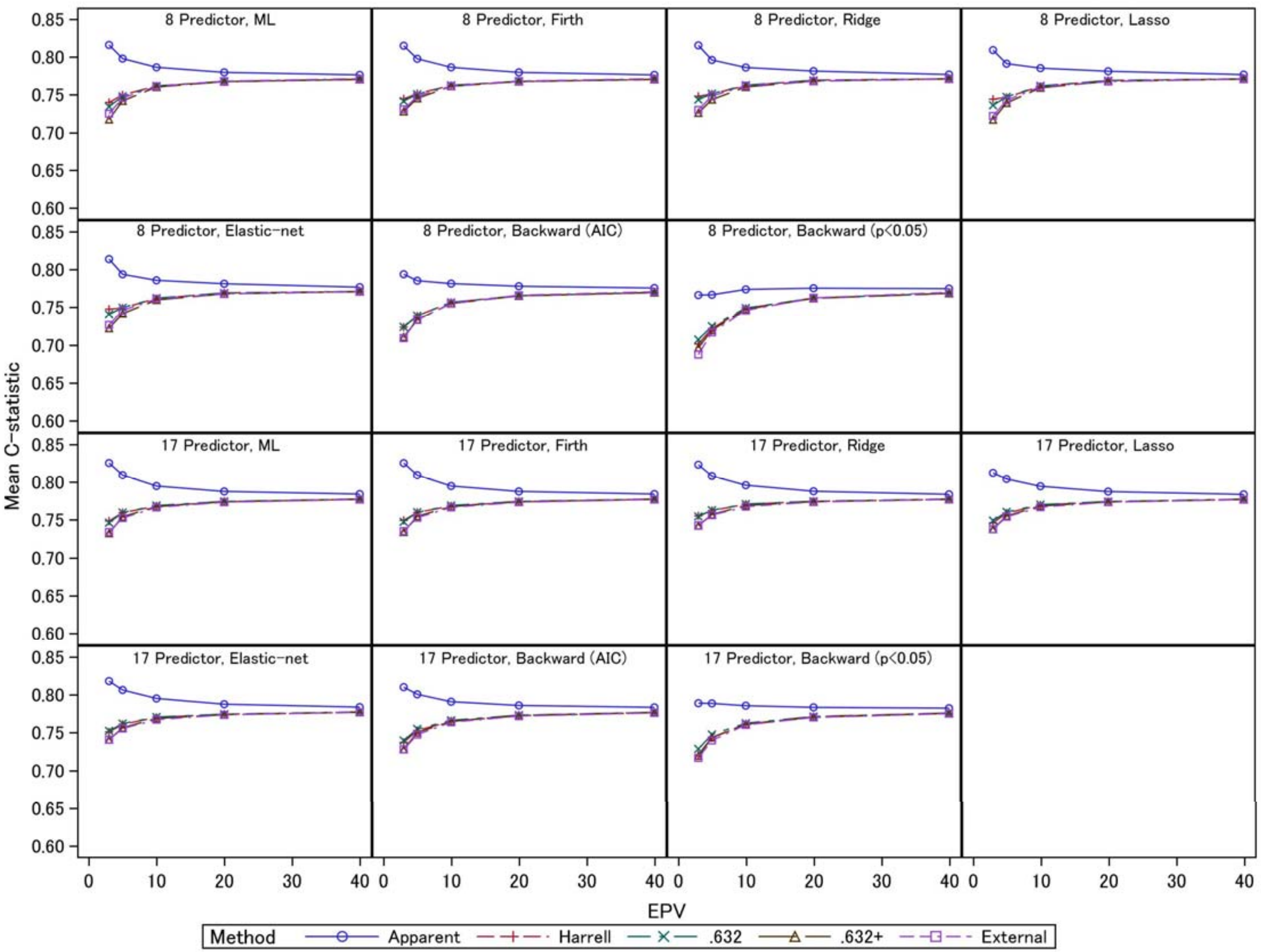

e-Figure 2. Simulation results: apparent, external, and optimism corrected *C*-statistics (scenario 1 and event fraction = 0.25)

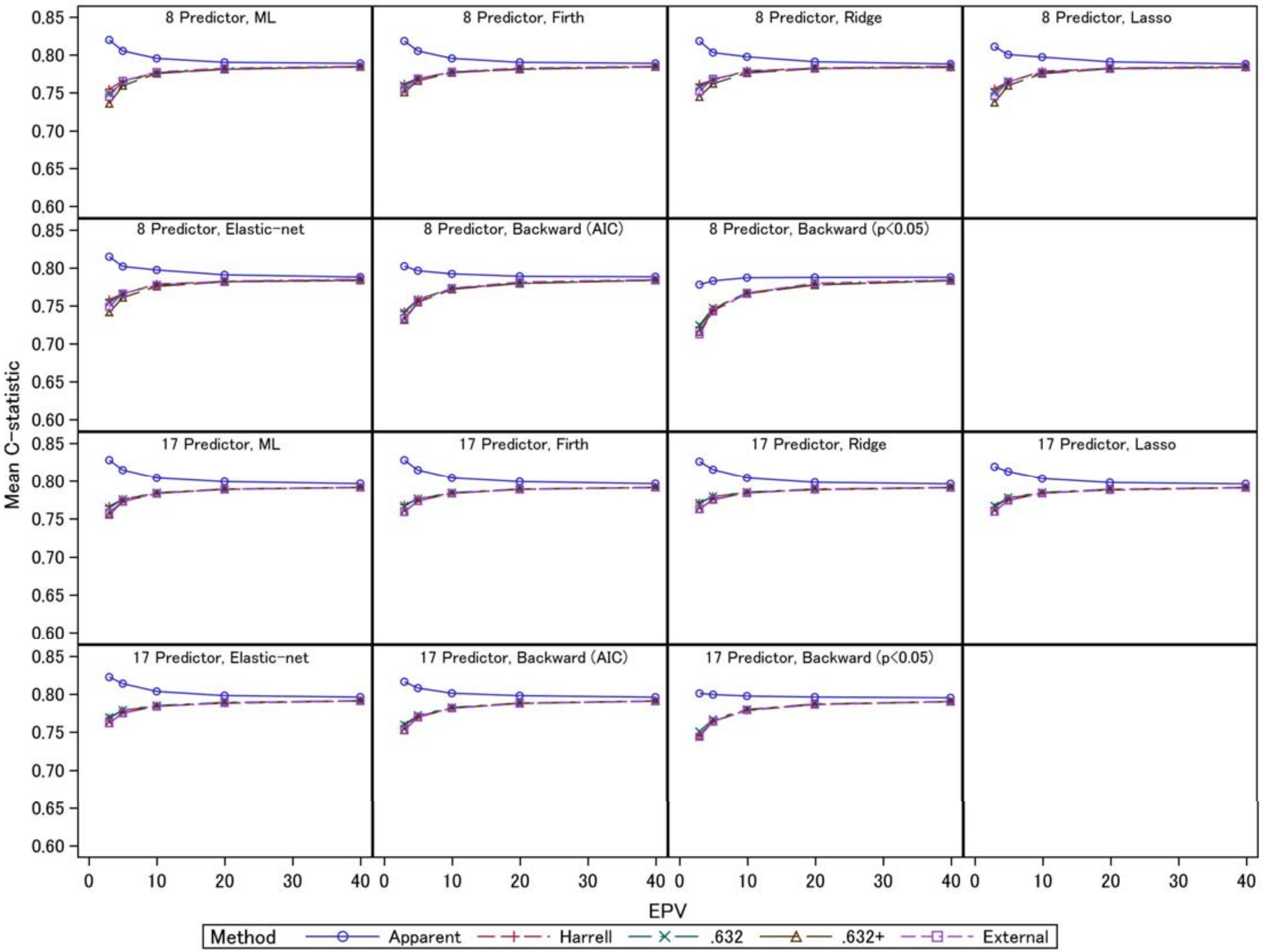

e-Figure 3. Simulation results: apparent, external, and optimism corrected *C*-statistics (scenario 1 and event fraction = 0.125)

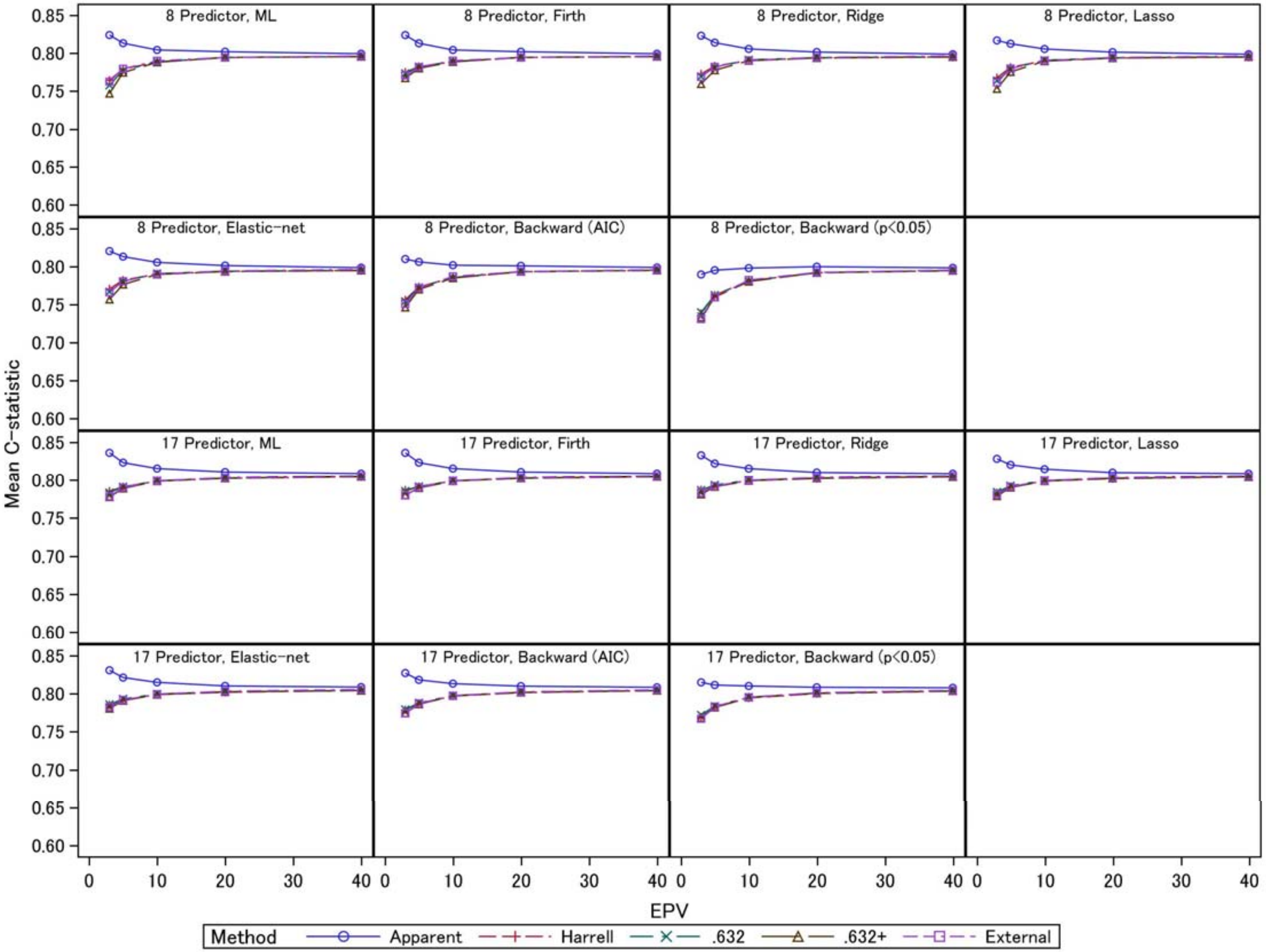

e-Figure 4. Simulation results: apparent, external, and optimism corrected *C*-statistics (scenario 1 and event fraction = 0.0625)

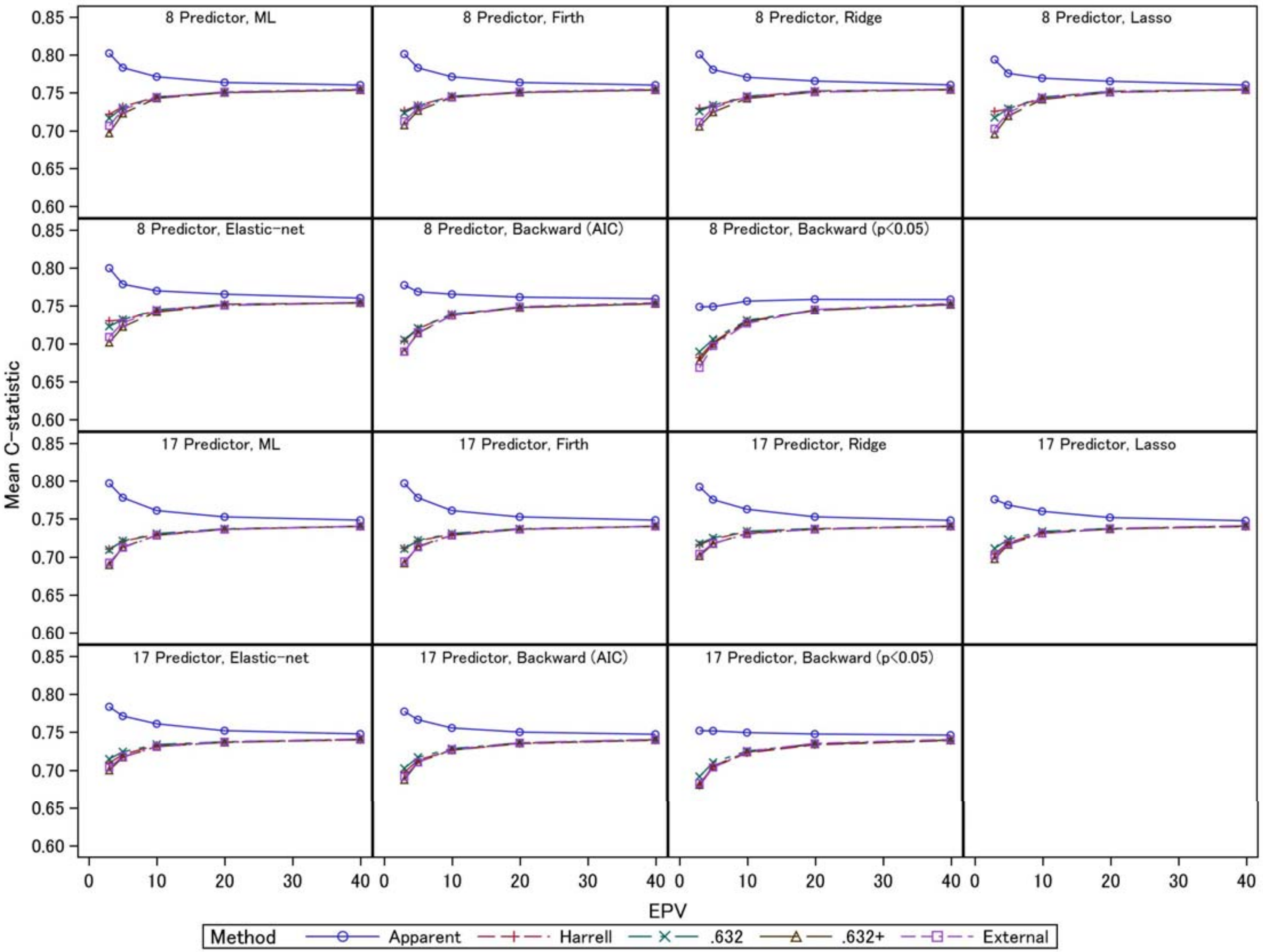

e-Figure 5. Simulation results: apparent, external, and optimism corrected *C*-statistics (scenario 2 and event fraction = 0.25)

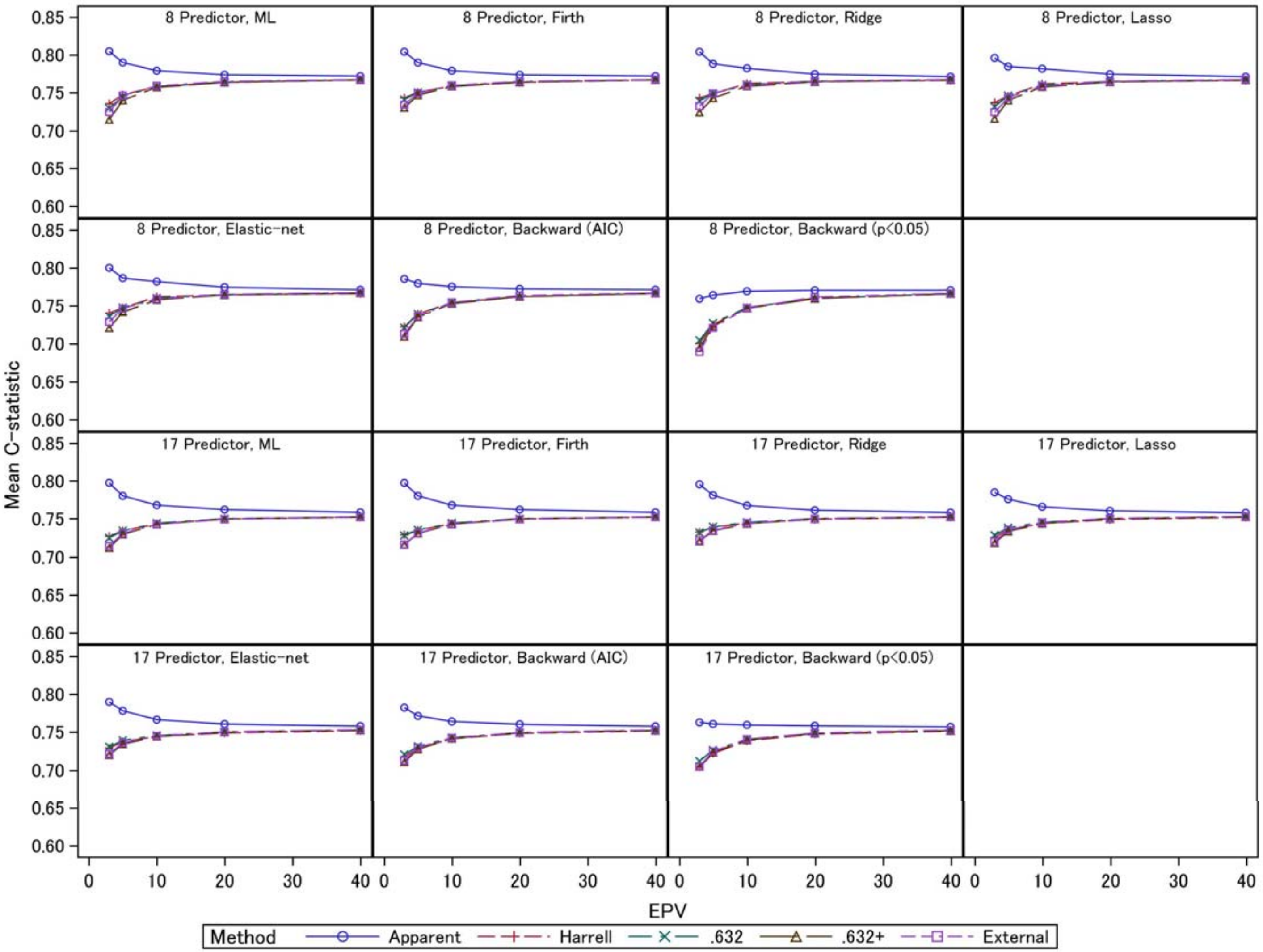

e-Figure 6. Simulation results: apparent, external, and optimism corrected *C*-statistics (scenario 2 and event fraction = 0.125)

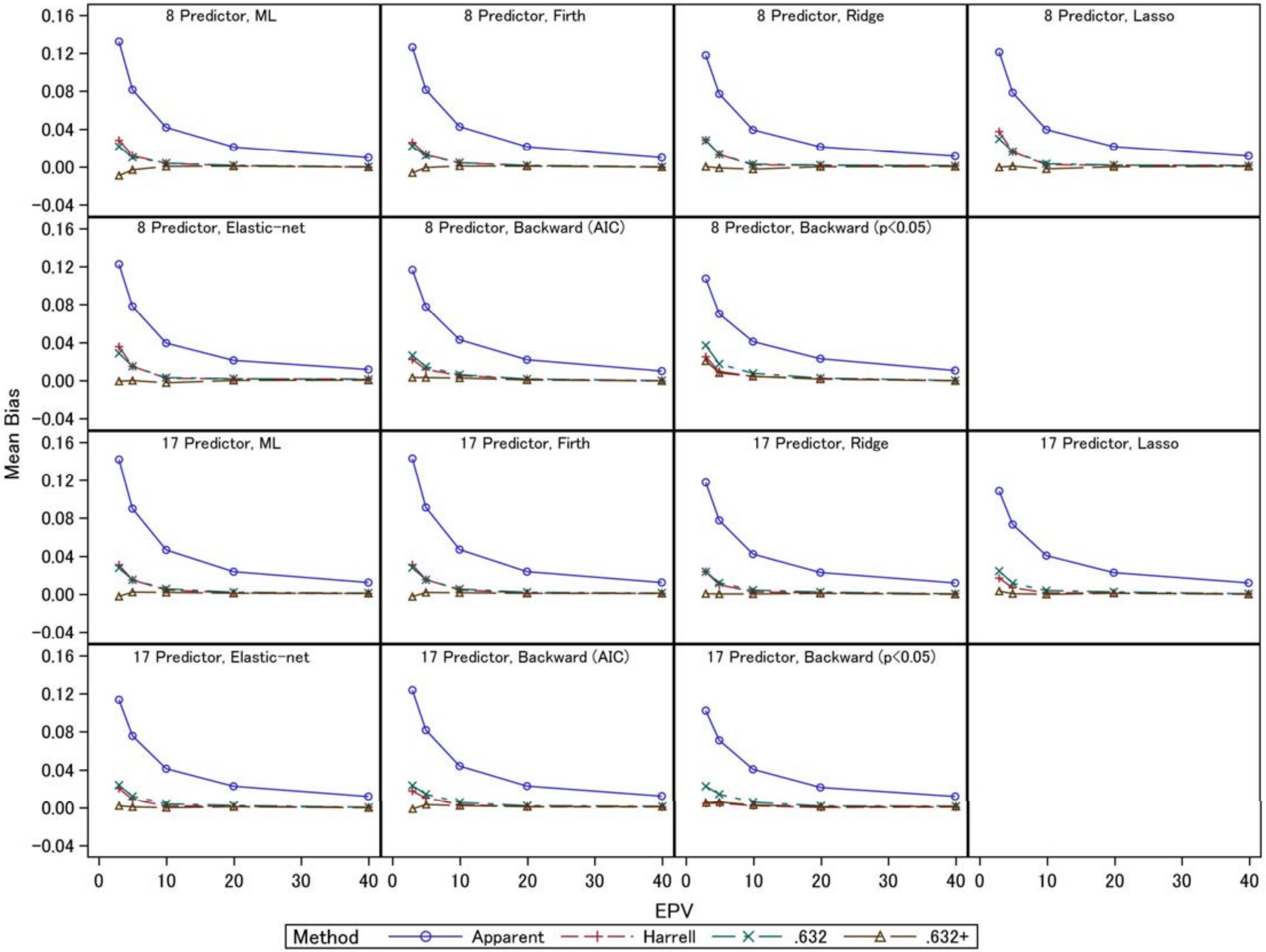

e-Figure 7. Simulation results: bias in apparent and optimism corrected *C*-statistics (scenario 1 and event fraction = 0.5)

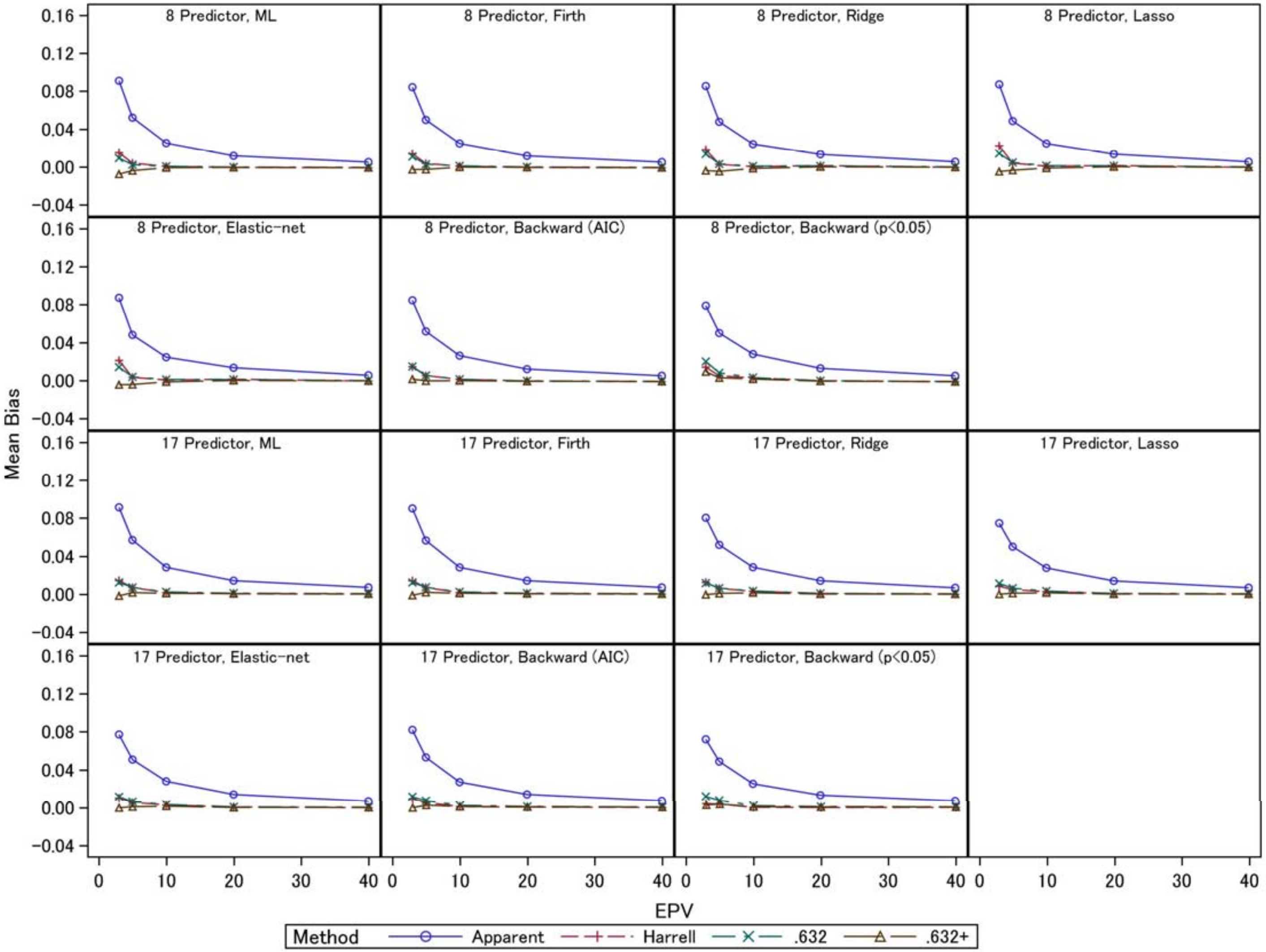

e-Figure 8. Simulation results: bias in apparent and optimism corrected *C*-statistics (scenario 1 and event fraction = 0.25)

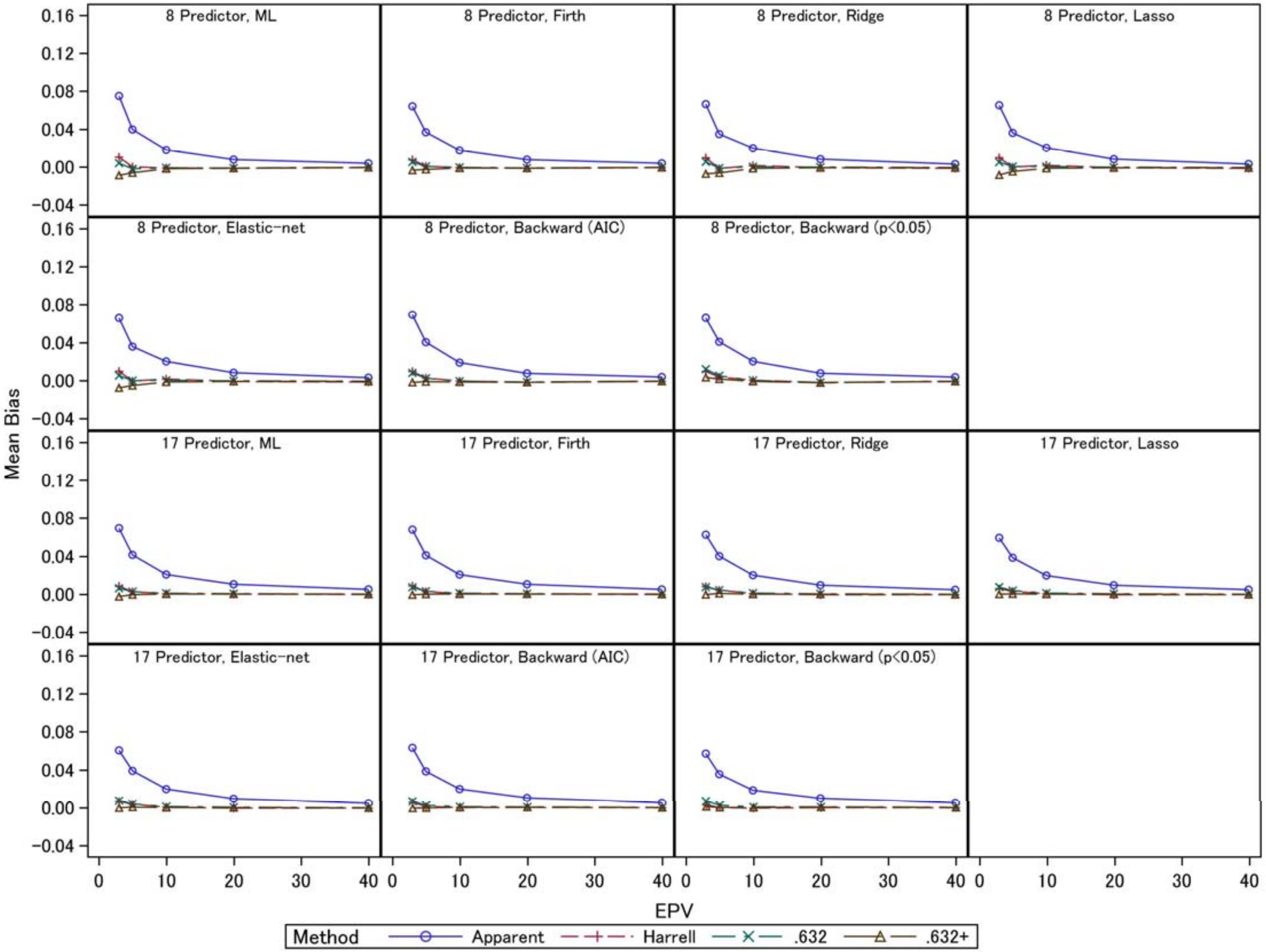

e-Figure 9. Simulation results: bias in apparent and optimism corrected *C*-statistics (scenario 1 and event fraction = 0.125)

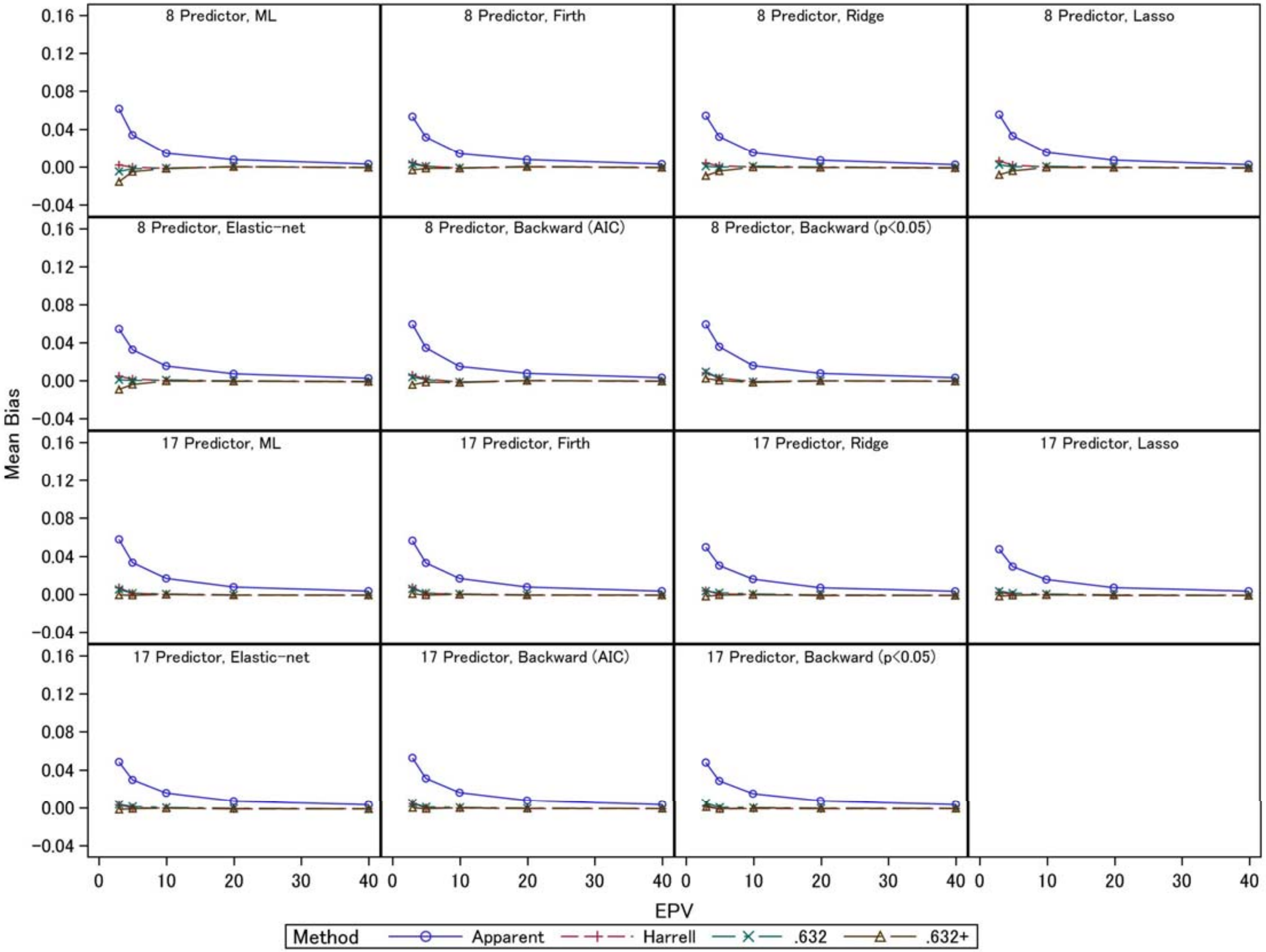

e-Figure 10. Simulation results: bias in apparent and optimism corrected *C*-statistics (scenario 1 and event fraction = 0.0625)

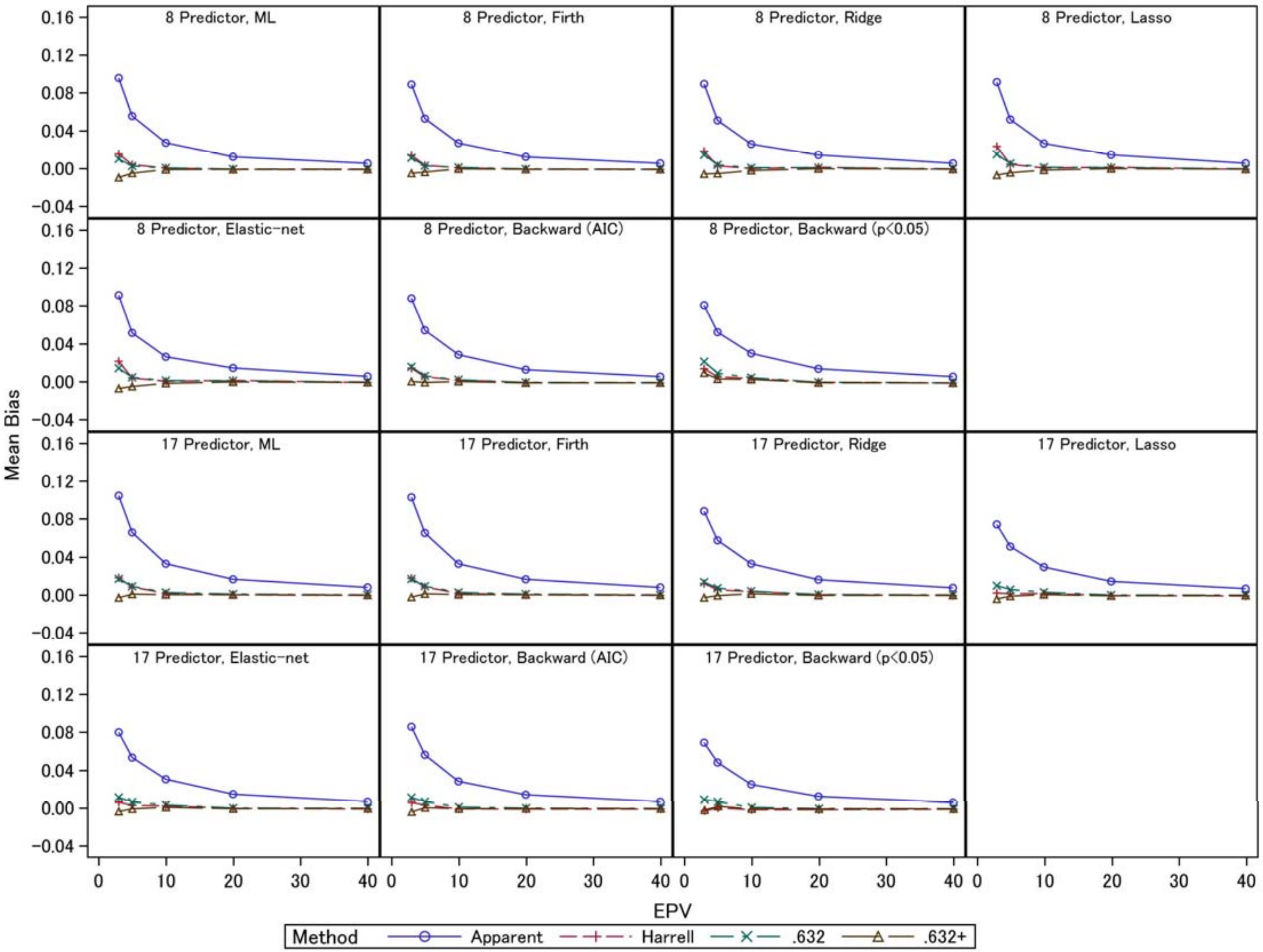

e-Figure 11. Simulation results: bias in apparent and optimism corrected *C*-statistics (scenario 2 and event fraction = 0.25)

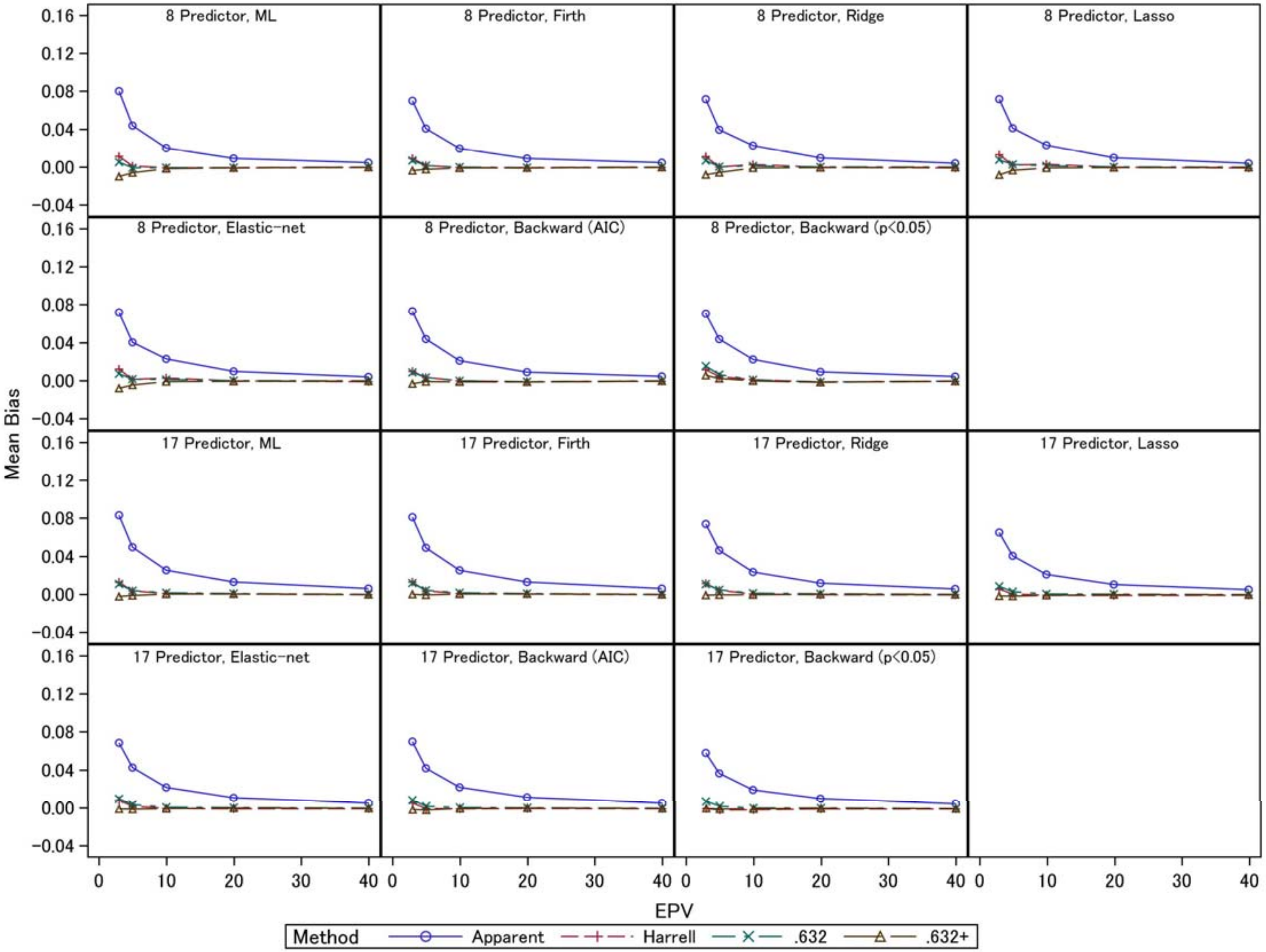

e-Figure 12. Simulation results: bias in apparent and optimism corrected *C*-statistics (scenario 2 and event fraction = 0.125)

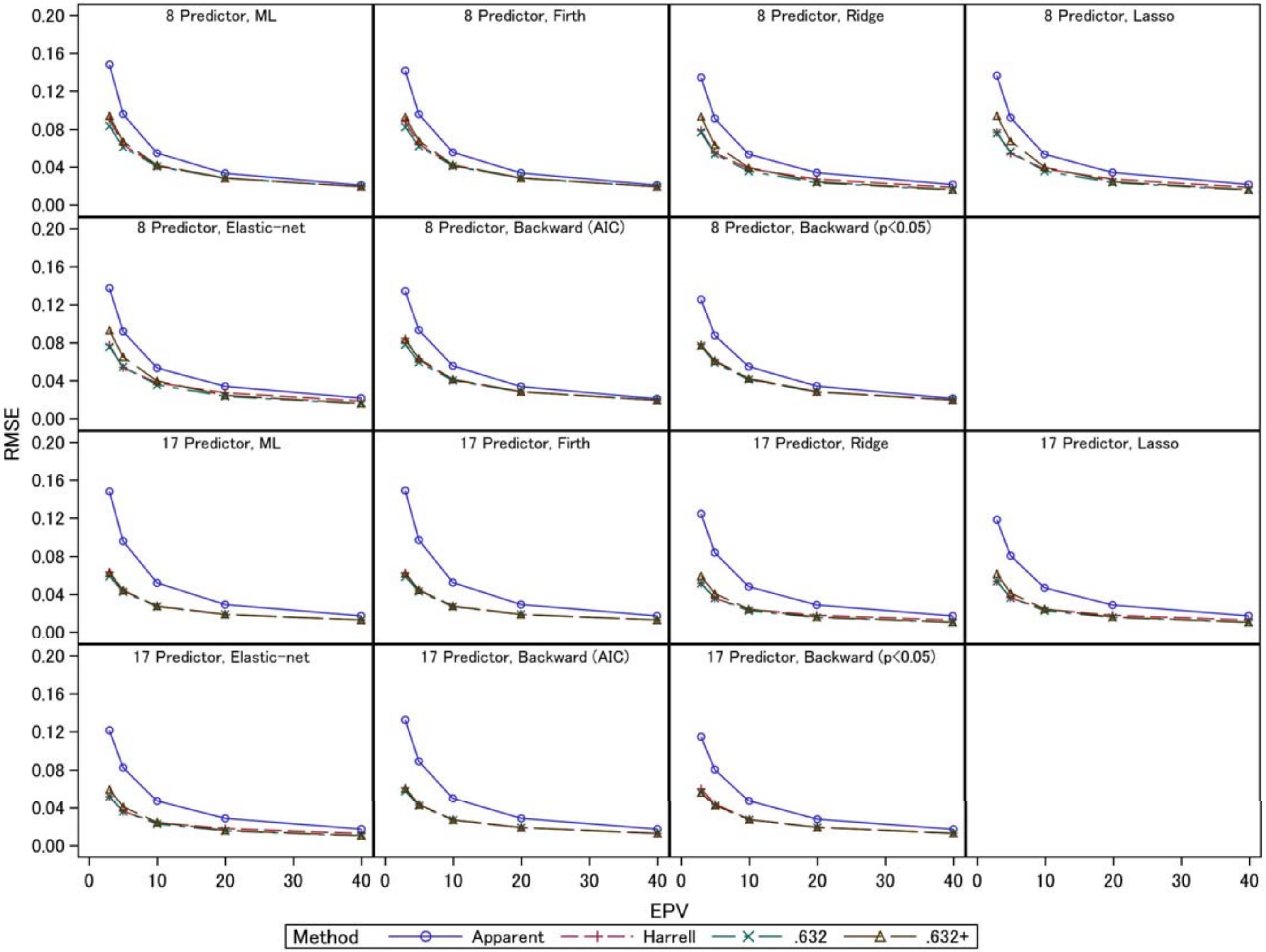

e-Figure 13. Simulation results: RMSE in apparent and optimism corrected *C*-statistics (scenario 1 and event fraction = 0.5)

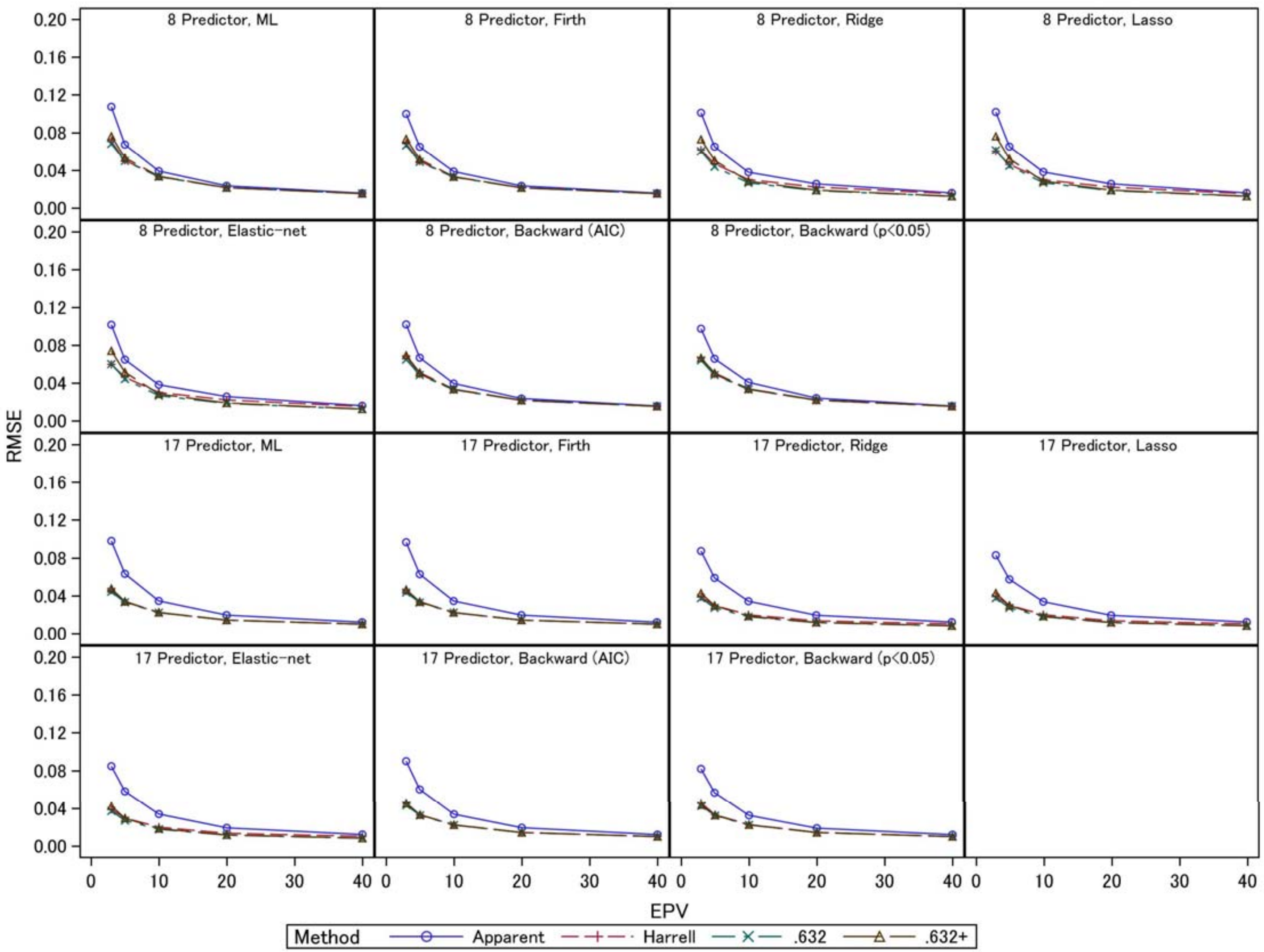

e-Figure 14. Simulation results: RMSE in apparent and optimism corrected *C*-statistics (scenario 1 and event fraction = 0.25)

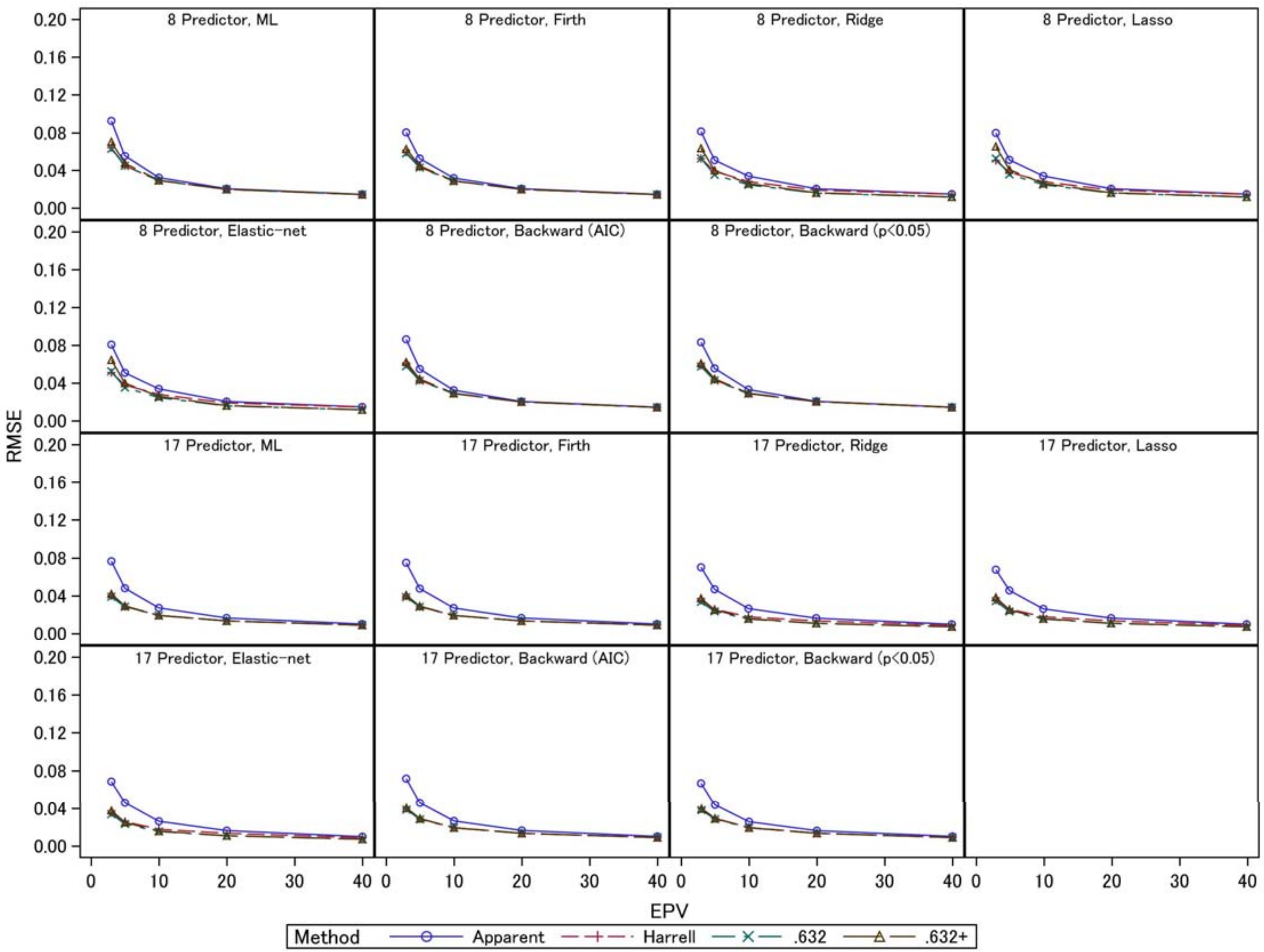

e-Figure 15. Simulation results: RMSE in apparent and optimism corrected *C*-statistics (scenario 1 and event fraction = 0.125)

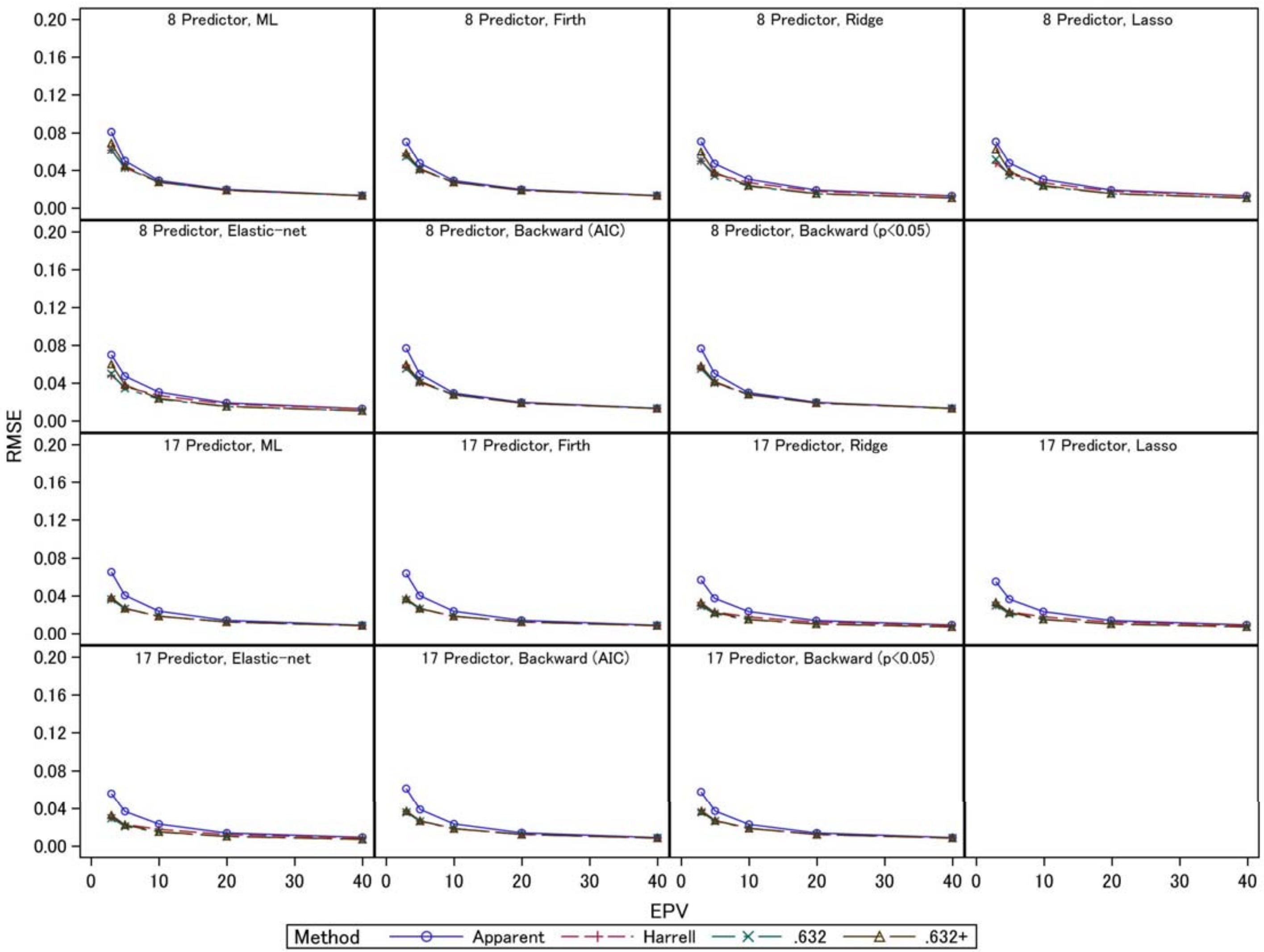

e-Figure 16. Simulation results: RMSE in apparent and optimism corrected *C*-statistics (scenario 1 and event fraction = 0.0625)

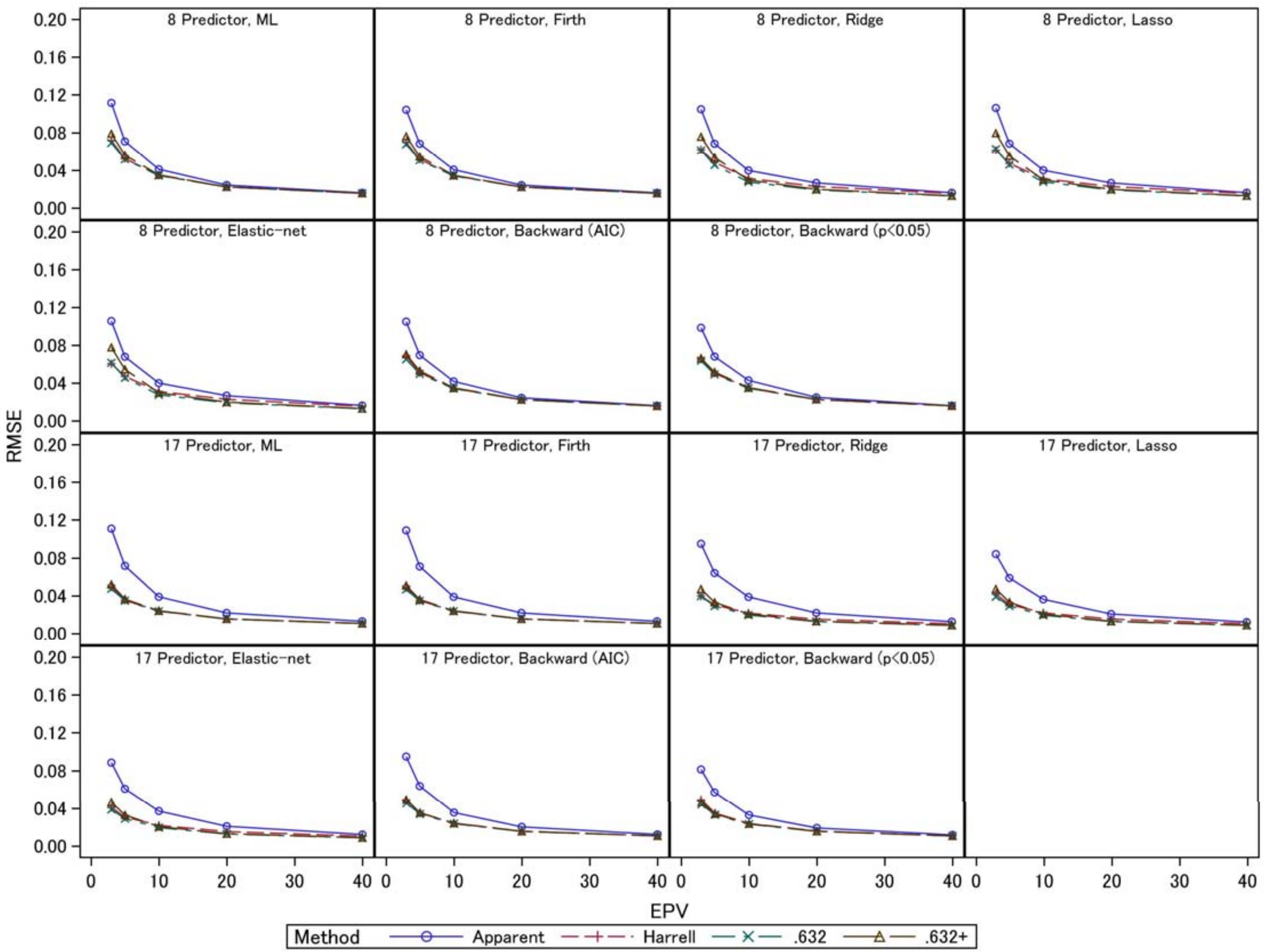

e-Figure 17. Simulation results: RMSE in apparent and optimism corrected *C*-statistics (scenario 2 and event fraction = 0.25)

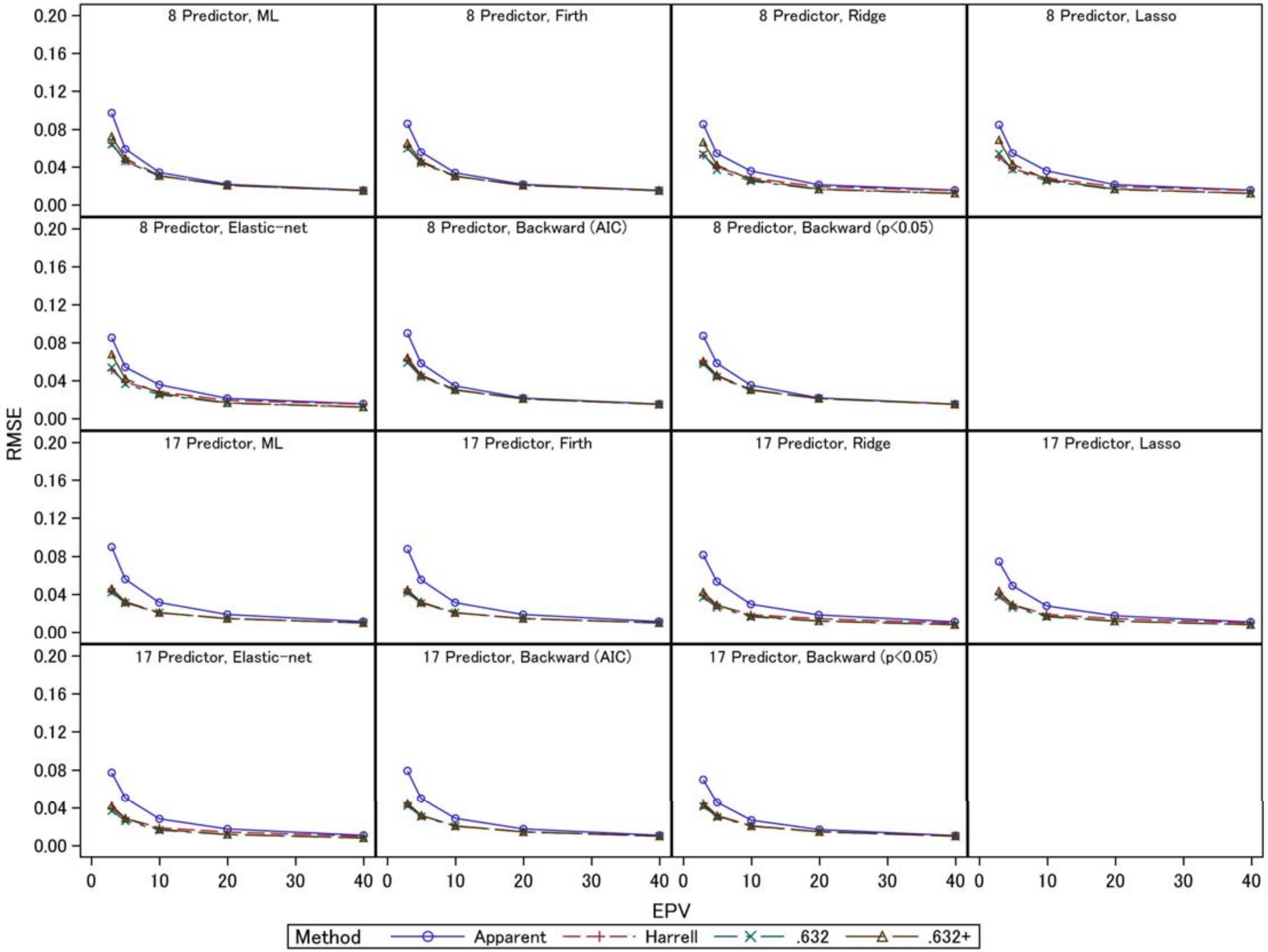

e-Figure 18. Simulation results: RMSE in apparent and optimism corrected *C*-statistics (scenario 2 and event fraction = 0.125)

e-Appendix B: Supplementary tables

In the Simulations section, we mentioned the final estimated models by lasso, elastic-net, and stepwise selections degenerated to only intercept models at certain frequencies. We present the actual proportions that the only intercept models were obtained from these variable selection methods in e-Table 1. The proportions of only intercept models were high for the 8-predictor models under EPV = 3 and event fraction = 0.5. Since these settings had the smallest sample size (N = 44) in the all settings, the variable selection methods (stepwise, lasso, and elastic-net) did not perform well sometimes. The high frequency of occurrences of the only-intercept model indicates that these variable selection methods cannot be used under such small sample settings sometimes. Even under these settings, Firth and ridge regressions can be used.

In addition, we discussed instabilities of the *C*-statistic estimators of lasso predictive models under small sample settings. It can be caused that the tuning parameter was selected by 10-fold CV, i.e., the small datasets were splitted to 10 subgroups, and the resultant individual training datasets can involve only small number of events. To assess the sensitivity of this estimating method, we conducted a supplementary simulations using leave-one-out CV. e-Table 2 show the supplementary results by lasso of the eight-predictor model at EPV = 3.

e-Table 1. Proportions (%) that the only intercept models obtained from the variable selection methods: lasso, elastic-net, stepwise selections †

		Lasso				Elastic-net				Stepwise selection (AIC)				Stepwise selection (p<0.05)			
	Events	8 Predictor		17 Predictor		8 Predictor		17 Predictor		8 Predictor		17 Predictor		8 Predictor		17 Predictor	
EPV	fraction	S1	S2	S1	S2	S1	S2	S1	S2	S1	S2	S1	S2	S1	S2	S1	S2
3	0.5	17.95	20.50	2.45	5.60	9.80	12.60	1.00	2.05	1.15	1.85	0.00	0.05	11.20	14.10	0.15	0.65
3	0.25	5.35	7.60	0.00	0.40	2.90	4.20	0.00	0.10	0.30	0.55	0.00	0.00	2.50	4.20	0.00	0.05
3	0.125	1.50	3.00	0.00	0.00	0.50	1.25	0.00	0.00	0.00	0.05	0.00	0.00	0.40	1.10	0.00	0.00
3	0.0625	1.05	1.30	0.00	0.10	0.40	0.35	0.00	0.00	0.05	0.15	0.00	0.00	0.25	0.65	0.00	0.00
5	0.5	3.80	5.70	0.15	0.50	1.90	3.10	0.00	0.00	0.15	0.30	0.00	0.00	2.25	3.80	0.00	0.00
5	0.25	0.85	1.35	0.00	0.00	0.40	0.90	0.00	0.00	0.00	0.00	0.00	0.00	0.20	0.45	0.00	0.00
5	0.125	0.25	0.55	0.00	0.00	0.15	0.25	0.00	0.00	0.00	0.00	0.00	0.00	0.05	0.10	0.00	0.00
5	0.0625	0.00	0.10	0.00	0.00	0.00	0.05	0.00	0.00	0.00	0.00	0.00	0.00	0.00	0.00	0.00	0.00
10	0.5	0.10	0.15	0.00	0.00	0.00	0.00	0.00	0.00	0.00	0.00	0.00	0.00	0.00	0.05	0.00	0.00
10	0.25	0.00	0.00	0.00	0.00	0.00	0.00	0.00	0.00	0.00	0.00	0.00	0.00	0.00	0.00	0.00	0.00
10	0.125	0.00	0.00	0.00	0.00	0.00	0.00	0.00	0.00	0.00	0.00	0.00	0.00	0.00	0.00	0.00	0.00
10	0.0625	0.00	0.00	0.00	0.00	0.00	0.00	0.00	0.00	0.00	0.00	0.00	0.00	0.00	0.00	0.00	0.00

There were no intercept model at EPV = 20 and EPV = 40.
† S1: Scenario 1, S2: Scenario 2

e-Table 2. RMSE in apparent and optimism corrected *C*-statistics for lasso of the eight-predictor model based on 10-fold and leave-one-out CV at EPV = 3

	Events	10-fold CV				Leave-one-out CV			
Scenario	fraction	Apparent	Harrell	.632	.632+	Apparent	Harrell	.632	.632+
1	0.0625	0.070	0.047	0.051	0.062	0.072	0.054	0.054	0.058
1	0.125	0.080	0.050	0.052	0.065	0.080	0.055	0.055	0.060
1	0.25	0.102	0.060	0.061	0.076	0.096	0.064	0.062	0.067
1	0.5	0.136	0.077	0.076	0.094	0.136	0.084	0.080	0.082
2	0.0625	0.075	0.048	0.054	0.067	0.076	0.054	0.055	0.059
2	0.125	0.085	0.050	0.054	0.069	0.086	0.057	0.057	0.061
2	0.25	0.106	0.061	0.062	0.079	0.101	0.065	0.063	0.067
2	0.5	0.145	0.080	0.079	0.096	0.142	0.086	0.082	0.083

e-Appendix C: Sensitivity analyses for skewed continuous variables settings

As mentioned in the Simulations section, we conducted sensitivity analyses for skewed continuous variables settings. We generated the continuous variables (height, weight, and age) from a multivariate skew normal distribution [36] with the parameters estimated from the GUSTO-I Western dataset instead of a multivariate normal distribution. The skewness parameters of the skew normal distribution [36] estimated from the GUSTO-I Western dataset for the height, weight, and age variables were −1.1, 3.2, and 0.0, respectively. The sensitivity analyses for the skewed variables settings were conducted only for the ML estimation.

The average values of the apparent, external, and optimism-corrected *C*-statistics are presented in e-Figure 19 (scenario 1) and e-Figure 20 (scenario 2). The empirical biases of the estimators of *C*-statistics derived from the external *C*-statistics are presented in e-Figure 21 (scenario 1) and e-Figure 22 (scenario 2). The empirical RMSEs are presented in e-Figure 23 (scenario 1) and e-Figure 24 (scenario 2). The simulation results based on multivariate normal and skew normal distributions were comparable in the all settings.

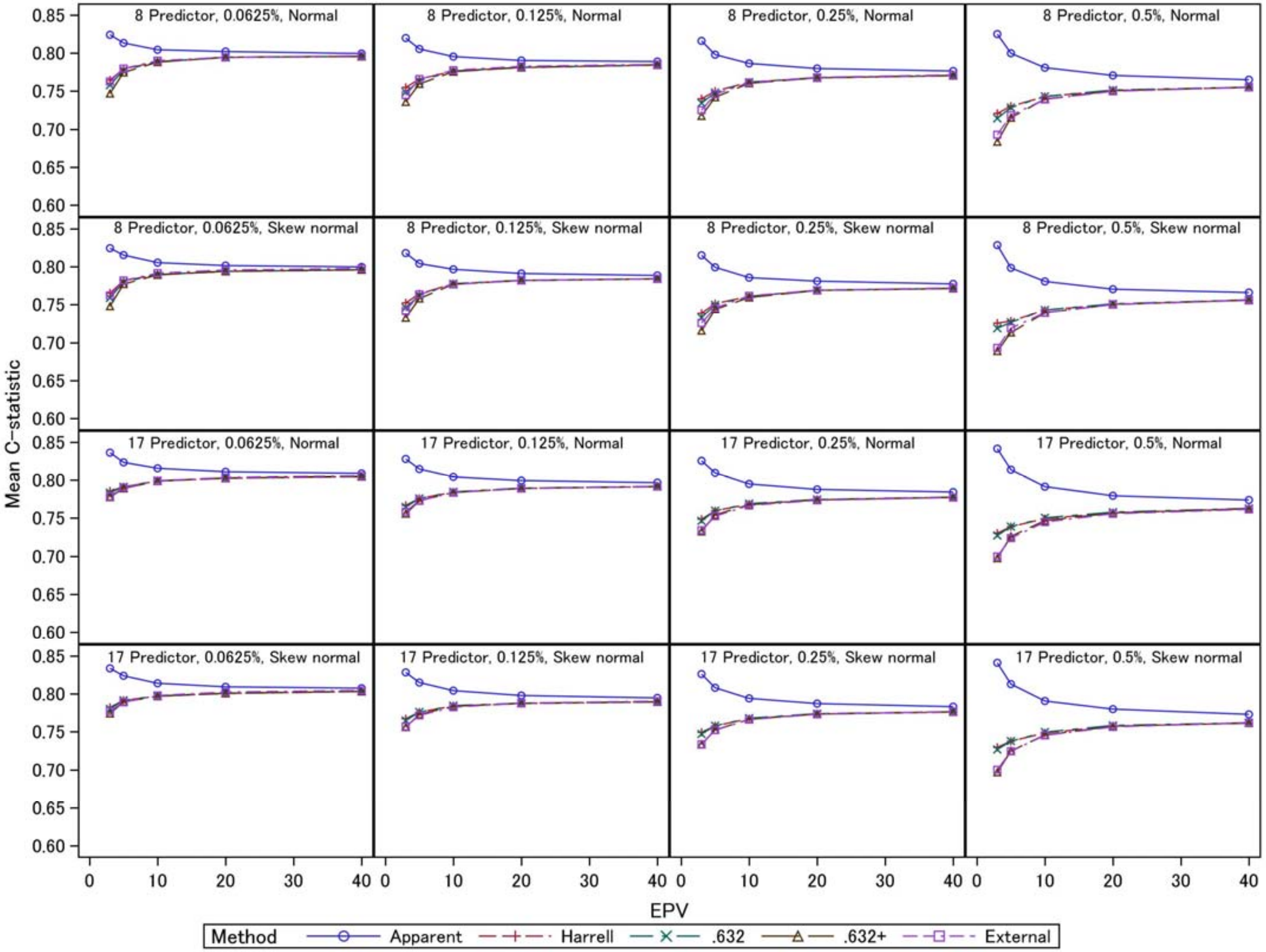

e-Figure 19. Simulation results based on multivariate normal and skew normal distributions: apparent, external, and optimism corrected *C*-statistics (scenario 1, ML estimation)

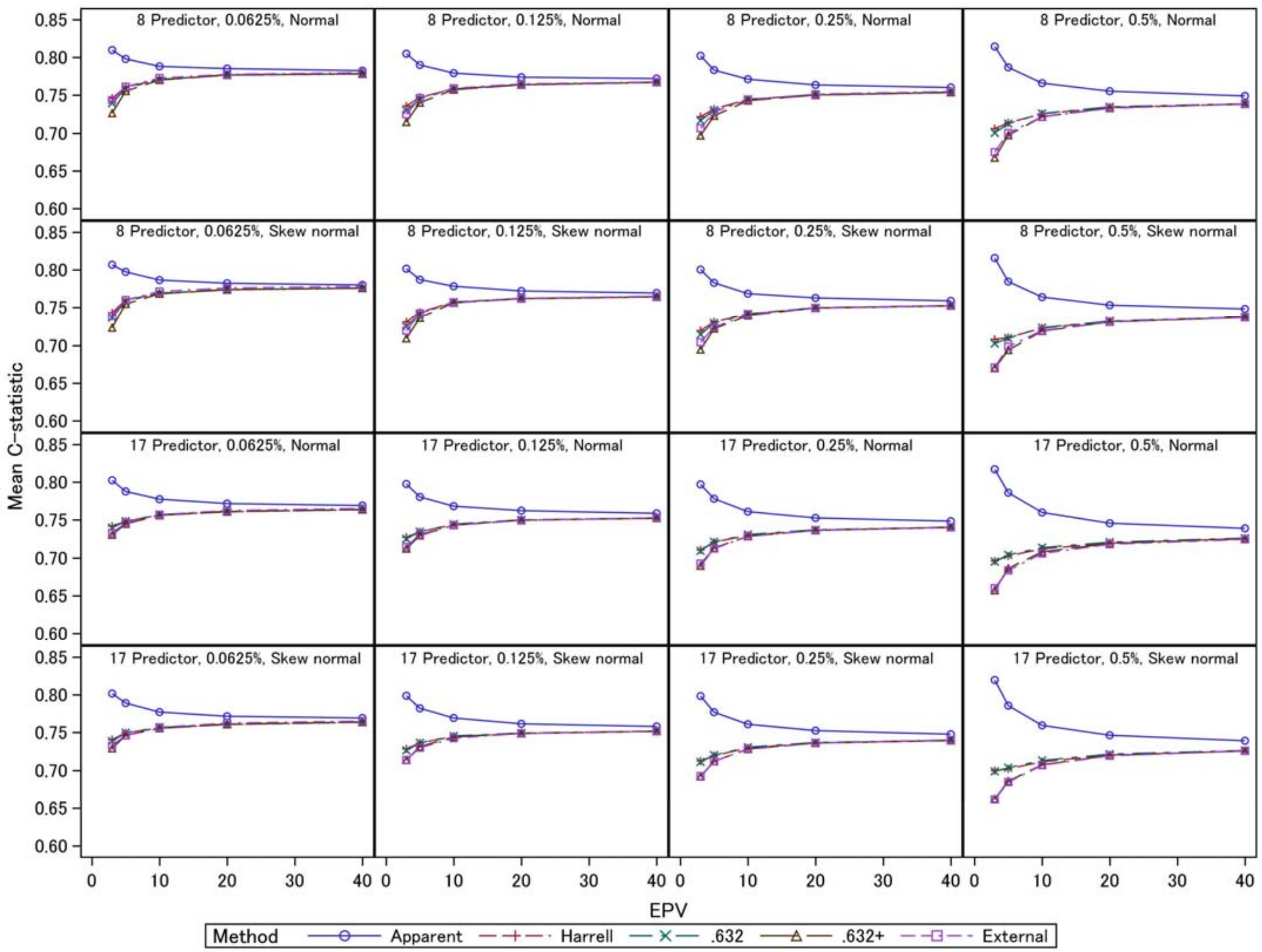

e-Figure 20. Simulation results based on multivariate normal and skew normal distributions: apparent, external, and optimism corrected *C*-statistics (scenario 2, ML estimation)

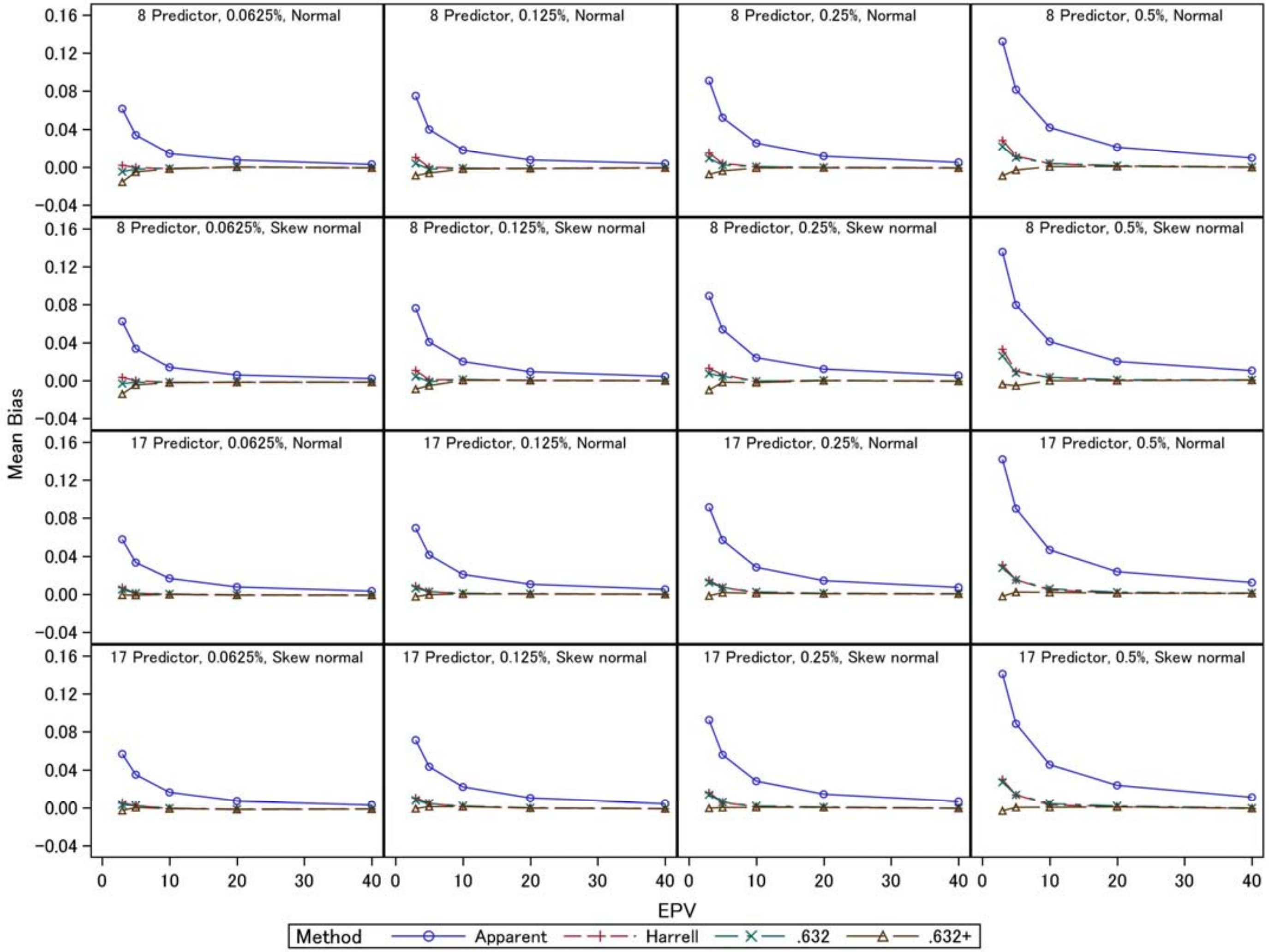

e-Figure 21. Simulation results based on multivariate normal and skew normal distributions: bias in apparent and optimism corrected *C*-statistics (scenario 1, ML estimation)

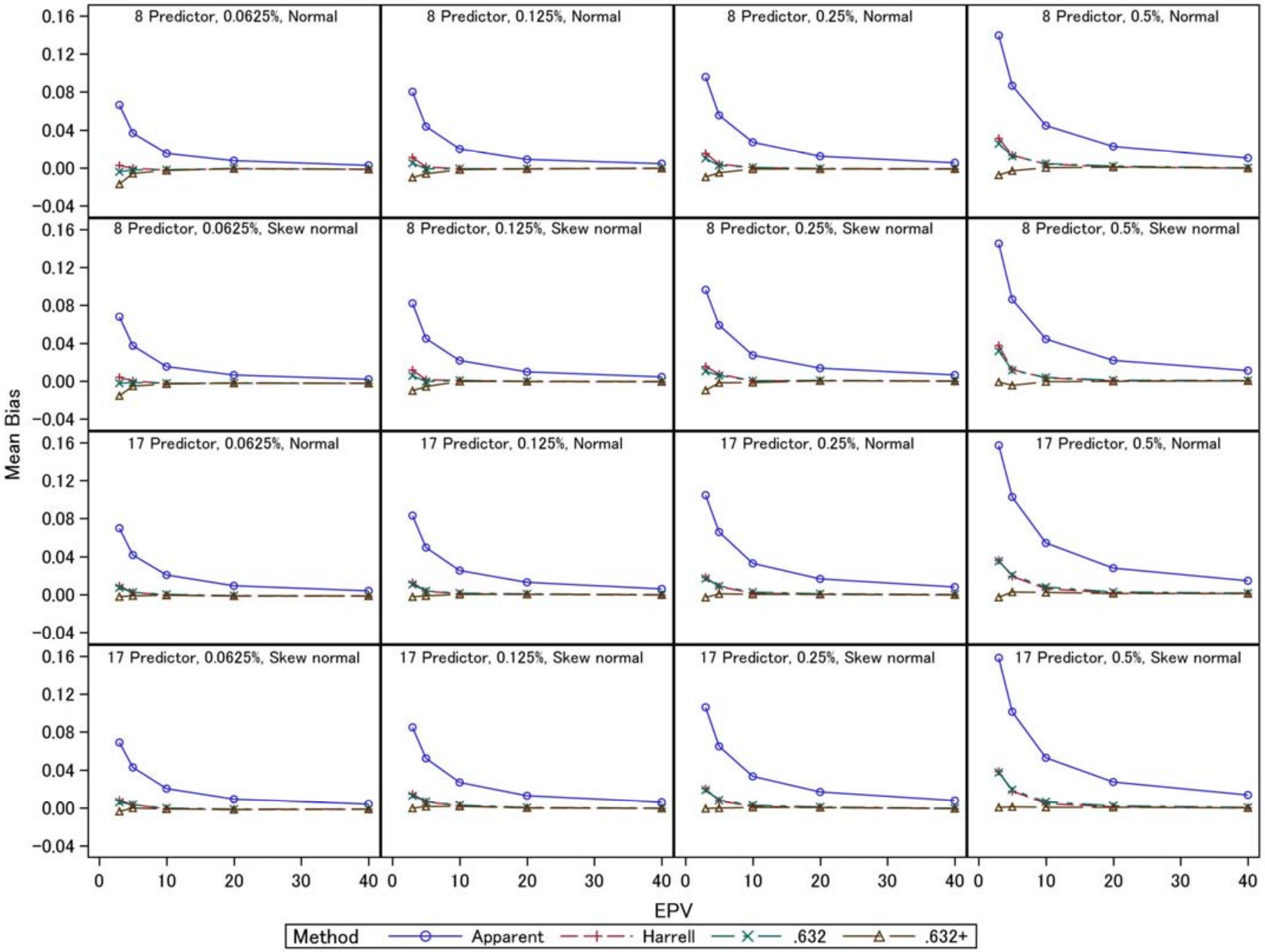

e-Figure 22. Simulation results based on multivariate normal and skew normal distributions: bias in apparent and optimism corrected *C*-statistics (scenario 2, ML estimation)

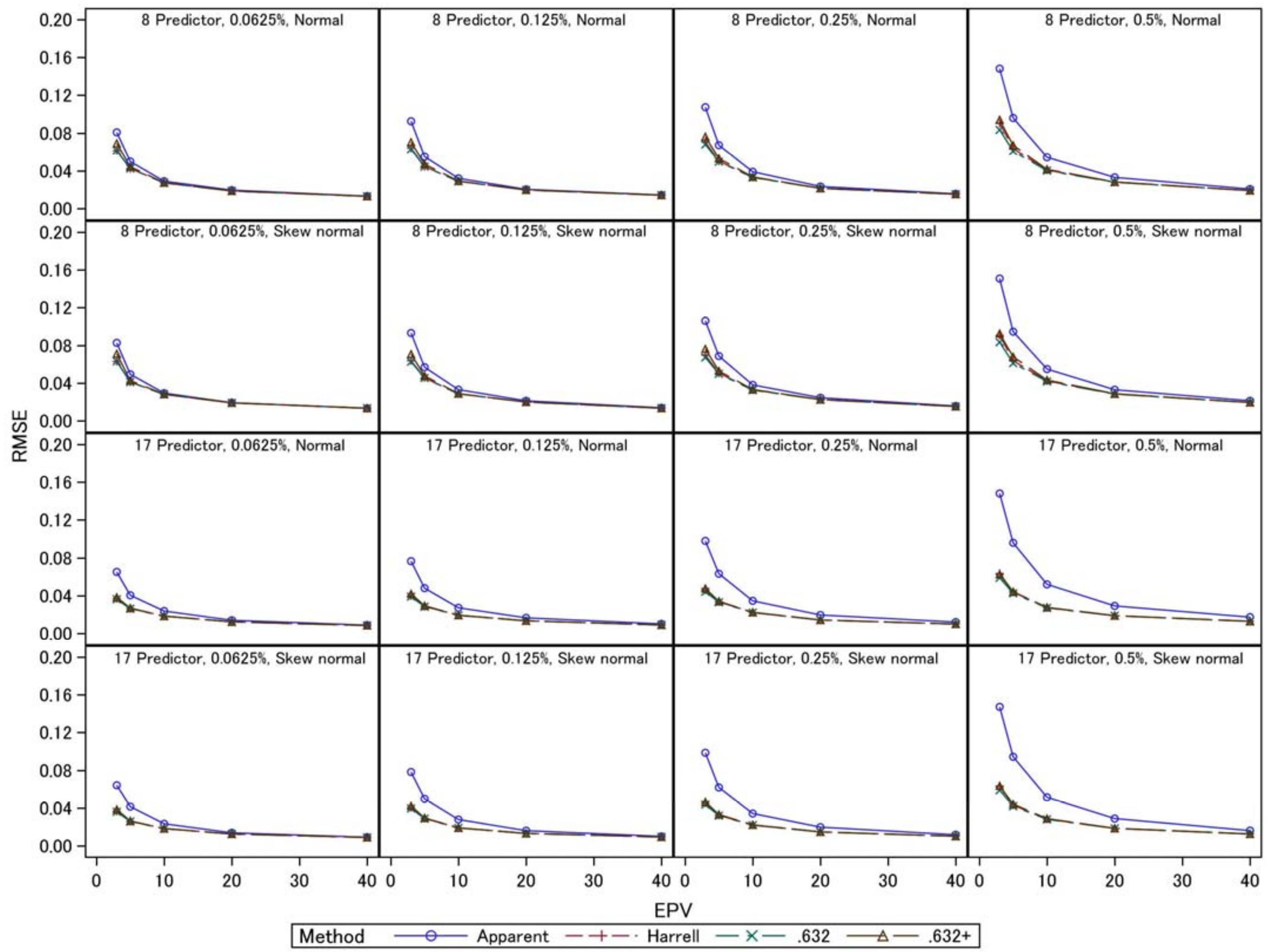

e-Figure 23. Simulation results based on multivariate normal and skew normal distributions: RMSE in apparent and optimism corrected *C*-statistics (scenario 1, ML estimation)

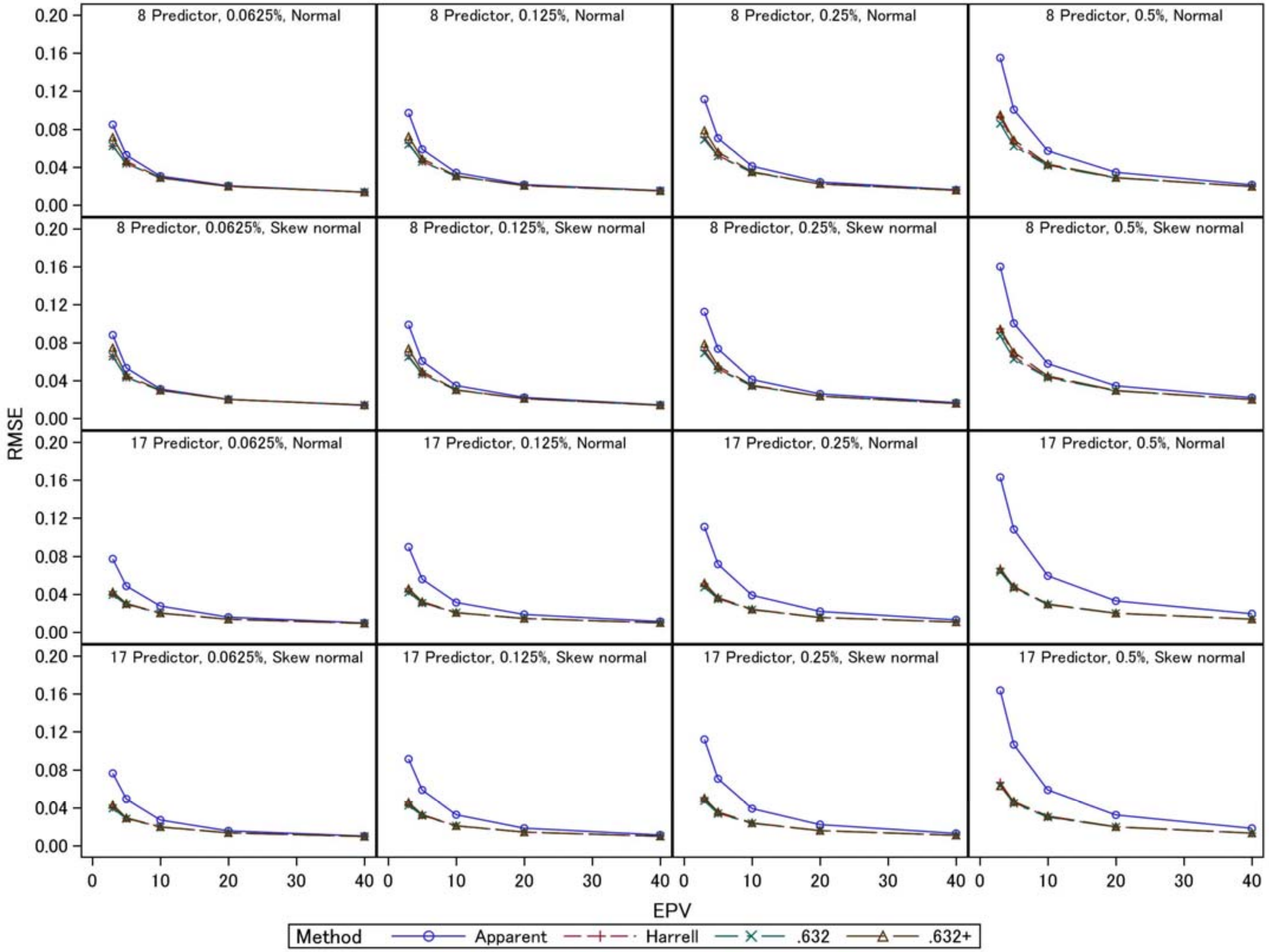

e-Figure 24. Simulation results based on multivariate normal and skew normal distributions: RMSE in apparent and optimism corrected *C*-statistics (scenario 2, ML estimation)
